# *CERKL*-Associated Retinal Dystrophy: Genetics, Phenotype, and Natural History

**DOI:** 10.1016/j.oret.2023.06.007

**Published:** 2023-10

**Authors:** Malena Daich Varela, Emma S. Duignan, Samantha R. De Silva, Rola Ba-Abbad, Yu Fujinami-Yokokawa, Shaun Leo, Kaoru Fujinami, Omar A. Mahroo, Anthony G. Robson, Andrew R. Webster, Michel Michaelides

**Affiliations:** 1 Moorfields Eye Hospital, London, United Kingdom; 2 UCL Institute of Ophthalmology, University College London, London, United Kingdom; 3 Royal Victoria Eye and Ear Hospital, Dublin, Ireland; 4 Ocular Genetics Services, King Khaled Eye Specialist Hospital, Riyadh, Saudi Arabia; 5 Laboratory of Visual Physiology, Division of Vision Research, National Institute of Sensory Organs, National Hospital Organization Tokyo Medical Center, Tokyo, Japan; 6 Department of Health Policy and Management, School of Medicine, Keio University, Tokyo, Japan

**Keywords:** Cone-rod, Dystrophy, Genetics, Inherited, Rod-cone

## Abstract

**Purpose:**

To analyze the clinical characteristics, natural history, and genetics of *CERKL*-associated retinal dystrophy in the largest series to date.

**Design:**

Multicenter retrospective cohort study.

**Subjects:**

Forty-seven patients (37 families) with likely disease-causing *CERKL* variants.

**Methods:**

Review of clinical notes, ophthalmic images, and molecular diagnosis from 2 international centers.

**Main outcome measures:**

Visual function, retinal imaging, and characteristics were evaluated and correlated.

**Results:**

The mean age at the first visit was 29.6 ± 13.9 years, and the mean follow-up time was 9.1 ± 7.4 years. The most frequent initial symptom was central vision loss (40%), and the most common retinal feature was well-demarcated areas of macular atrophy (57%). Seventy-seven percent of the participants had double-null genotypes, and 64% had electrophysiological assessment. Among the latter, 53% showed similar severity of rod and cone dysfunction, 27% revealed a rod-cone, 10% a cone-rod, and 10% a macular dystrophy dysfunction pattern. Patients without double-null genotypes tended to have fewer pigment deposits and included a higher proportion of older patients with a relatively mild electrophysiological phenotype. Longitudinal analysis showed that over half of the cohort lost 15 ETDRS letters or more in ≥ 1 eye during the first 5 years of follow-up.

**Conclusions:**

The phenotype of *CERKL*-retinal dystrophy is broad, encompassing isolated macular disease to severe retina-wide involvement, with a range of functional phenotypes, generally not fitting in the rod-cone/cone-rod dichotomy. Disease onset is often earlier, with more severe retinal degenerative changes and photoreceptor dysfunction, in nullizygous cases.

**Financial Disclosure(s):**

Proprietary or commercial disclosure may be found in the Footnotes and Disclosures at the end of this article.

Inherited retinal diseases (IRDs) are a group of heterogeneous stationary or progressive retinal disorders that often cause visual impairment.^1^ Inherited retinal diseases represent a leading cause of visual disability in the working-age population in the UK.^2^ Depending on whether rod or cone photoreceptors are primarily (or more severely) affected, 2 main phenotypes of IRD can be differentiated, cone-rod dystrophy (CORD) and rod-cone dystrophy (RCD); the latter also known as retinitis pigmentosa (RP). Macular dystrophy (MD) corresponds to an IRD where dysfunction is confined to the macula. Halting the progression of these conditions and alleviating their symptoms is the subject of multiple avenues of research.

Over 300 genes and loci are currently known to cause IRD (https://web.sph.uth.edu/RetNet/). Ceramide kinase-like (*CERKL*, MIM ∗608381) is among the rare causative genes in the UK, detected only in 17 families of a large cohort of over 3000 genetically diagnosed IRD families by 2020.^3^ It is also reported to be an uncommon cause of CORD in a German cohort.^4^ Other groups, however, report a higher prevalence of *CERKL*-associated retinopathy, accounting for 33% and 5% of autosomal recessive IRD in Yemenite Jewish and Spanish populations, respectively.^5^^,^^6^ *CERKL* was associated with 8% of IRD cases in Tunisia^7^ and was one of the most common genes in a large Spanish RP cohort study.^8^ To date, 4 founder, disease-causing variants have been described: NM_001030311.2: c.847C>T (p.Arg283Ter) (reported as NM_201548.5: c.769C>T) from Spain, c.238+1G>A from the Yemenite Jewish population, c.375C>G (p.Cys125Trp) from the Finnish, and c.365T>G (p.Leu122Arg) from indigenous African populations in South Africa.^5^^,^^9^, ^10^, ^11^

*CERKL* was first associated with IRD in 2004, after Tuson et al analyzed RP26, a 17.4-Mb locus in chromosome 2 known to cause RP in Spanish families.^9^ It is a 13-exon gene that encodes a 532-amino acid protein, with a diacylglycerol kinase and a pleckstrin homology domains.^12^ It is widely expressed in the kidney, lung, skin, and pancreas, and it is known to have a protective role against oxidative stress-induced apoptosis.^13^ It also interferes with mitochondrial metabolism, stress granules, and autophagy regulation, yet its pathophysiology remains to be fully elucidated.^14^

Biallelic loss-of-function variants in *CERKL* have been associated with both RCD and CORD.^15^^,^^16^ However, the reported retinal phenotype in both cases appears to be somewhat similar: a widespread retinopathy with early maculopathy and a similar severity of rod and cone dysfunction electrophysiologically.^5^^,^^15^ Patients often report concurrent cone- (e.g., central vision and color discrimination impairment) and rod-related (e.g., nyctalopia, peripheral field loss) symptoms, becoming noticeable from adolescence/young adulthood, with an unclear timeline.^17^ *CERKL* can also present with a Stargardt-like phenotype, often being a differential diagnosis when no variants are found in *ABCA4*. Nevertheless, there remains a paucity of detailed phenotypic characterization in a large cohort, thereby limiting our understanding of *CERKL*-associated retinopathy and its natural history. This study aims to establish the phenotypic and genetic spectrum by examining the largest case series of *CERKL*-associated retinopathy patients to date, to better understand this disorder and to optimize future clinical management.

## Methods

This study was a retrospective, consecutive case series of patients who attended Moorfields Eye hospital (MEH, London, UK) and the Royal Victoria Eye and Ear Hospital (Dublin, Ireland) with a retinal dystrophy and were found to have likely disease-causing variants in *CERKL*. At MEH, patients were identified through the inherited eye disease database. Informed consent was obtained from all patients. Ethical approval was provided by the local ethics committees, and the study honored the tenets of the Declaration of Helsinki.

Relevant patient data were retrieved from electronic healthcare records, case notes, and imaging software systems. Snellen visual acuities were recorded and converted to logarithm of the minimum angle of resolution (logMAR) for the purpose of statistical analysis. Logarithm of the minimum angle of resolution values were assigned as follows: count fingers vision logMAR 1.98, hand motion logMAR 2.28, light perception logMAR 2.7, and no light perception logMAR 3.^18^ Asymmetric best-corrected visual acuity (BCVA) was defined as a difference ≥ 0.3 logMAR (equivalent to 15 ETDRS letters) between eyes. Patients were categorized using the World Health Organization (WHO) visual impairment criteria, which define no or mild visual impairment as BCVA ≤ 0.48 (6/18, 20/60), moderate impairment as BCVA > 0.48 and ≤ 1.0 (6/60, 20/200), severe as BCVA >1.0 and ≤ 1.3 (3/60, 20/400), and blindness as BCVA > 1.3. Records of visual field were limited within our cohort; therefore, we classified patients according to BCVA only. “Low-vision” corresponds to patients with moderate and severe visual impairment.

Further clinical assessment consisted of dilated fundus examination, spectral-domain OCT (SD-OCT, Heidelberg Spectralis, Heidelberg Engineering, Inc), fundus autofluorescence (Heidelberg Spectralis, Heidelberg Engineering, Inc and Optos PLC), and ultrawide-field (UWF) fundus pseudocolor photography (Optos PLC). OCT thickness in the general population was extracted from Invernizzi et al.^19^ Fovea-centered macular volume scans were performed in a 6-mm^2^ area that included the 1-, 3-, and 6-mm grid templates from the ETDRS. Inner limiting membrane and Bruch membrane were automatically segmented by the manufacturer software (Heyex version 1.9.14.0; Heidelberg Engineering) and adjusted manually as needed by a trained ophthalmologist (M.D.V.). Ellipsoid zone (EZ) width and outer nuclear layer thickness (ONLT) were measured manually at the foveal scan. Patients with macular cysts, edema, and/or only line scans were excluded from quantitative assessment. Non-ocular issues were defined as those commonly found in syndromic IRDs, such as musculoskeletal, renal, cardiac, audiologic, or neuro/psychological.^20^

Pattern (PERG) and full-field (ERG) electroretinogram testing was performed incorporating the International Society for Clinical Electrophysiology of Vision (ISCEV) standards.^21^ Pattern ERG P50 was used as an objective measure of macular function and full-field ERG was used to assess generalized (mainly peripheral) rod and cone system function. The main dark-adapted (DA) and light-adapted (LA) ERG components were quantified and compared with age-matched control data from healthy subjects (age range, 10–79 years).^22^ Electroretinogram amplitudes were plotted as a percentage of the age-matched lower limit of the reference range or as a difference from the age-matched upper limit of peak time. The reference limits were defined as lower 5th percentile for amplitude and 95th percentile for peak times. The full-field ERGs were classified into 4 groups based on the relative reduction of DA and LA ERGs: rod and cone photoreceptor dysfunction, rod-cone, cone-rod, or predominantly macular dysfunction (normal or near-normal ERGs). For the purposes of the electrophysiological analysis, a 33% minimum difference in the relative reduction of DA 10 ERG a-wave and LA 3 ERG b-wave was used to define rod-cone and cone-rod patterns of dysfunction. An additional patient (age, 5 years; ID, 41) was tested before his baseline ophthalmic assessment according to shorter PERG and ERG protocols using lower eyelid skin electrodes without mydriasis.

DNA was extracted from whole blood and genetic testing was performed using panel-based targeted next-generation sequencing (NGS), whole-exome sequencing, or whole-genome sequencing. Where appropriate, blood samples were taken from parents or siblings to confirm segregation of proposed variants (i.e., to determine if relatives carry 1 or both variants in 1 or 2 alleles). Other IRD genes were excluded based on coding variant analysis. The pathogenicity of the variants was determined by implementing the criteria of the American College of Medical Genetics (ACMG).^23^ Likely disease-causing variants correspond to pathogenic, likely pathogenic, and selected variants of uncertain significance (VUS), based on family history, phenotype, and/or if concurrent with a pathogenic/likely pathogenic variant. *In silico* molecular genetic analysis was performed for all detected *CERKL* variants (Genome reference; Hg38 Transcript; NM_001030311.2, ENST00000339098.9 or NM_201548.5, ENST00000410087.8), according to a previous publication.^24^ Genotype grouping was performed according to the presence of null variants (those assumed to result in a loss of function [nonsense, frameshift, splice-site alteration, and exon deletion]); a double-null (DN) genotype harboring multiple null variants (also known as “nullizygous”), and a non–double-null (NDN) genotype with 1 or no null variants. Clinical descriptions and parameters were compared between subgroups of patients with DN and NDN genotypes.

GraphPad Prism 8.0.2 (GraphPad Software, San Diego) was used for statistical analysis. The threshold of significance was set at *P* < 0.05. Linear regressions and *t* tests were used for assessment of parametric variables. When testing associations between age and ocular characteristics, only the right eye was considered to avoid clustering effect. Welch's *t* test variation was employed when the sample sizes were significantly different.

## Results

### Demographics, Phenotype, and Visual Acuity

Forty-seven patients were characterized from 37 pedigrees. Their clinical characteristics are listed in Tables 1 and S2 (available at www.ophthalmologyretina.org). Twenty-two individuals were female (47%) and 25 (53%) were male. The mean age ± standard deviation (SD) at the first visit was 29.6 ± 13.9 years (range, 6–67 years), and the median age was 25 years, with 5 participants (11%) having their first visit below the age of 16. The mean follow-up time of the cohort was 9.1 ± 7.4 years, and the mean age at their final visit was 39.2 ± 16.6 years. One patient was deaf and had an autosomal dominant family history of deafness; no other participant had non-ocular issues. Among the patients who reported their ethnicity (72%), 19 were of Asian origin, 13 were White from UK and Europe, and 2 were of African descent. Consanguinity was reported in 6 families, in which parents were first cousins.

Based on ophthalmic history and clinical examination, 26 patients were initially diagnosed with RP (RCD), 13 with CORD, and 8 with MD. The mean age of symptoms onset was 19.8 ± 9 years (median, 17; range, 6–47 years), with 16 patients below the age of 16. Nineteen patients (40%) described central vision loss as their initial symptom, 16 (34%) reported concurrent blurred central vision and nyctalopia, 5 (11%) reported night blindness first, and the remaining patients mentioned being initially troubled by scotomas, photophobia, or poor color discrimination. A prolonged prodromal history of subtle visual acuity (VA) decrease was elicited in 6 patients (24%), lasting between 1 and 10 years.

**Figure 1:**
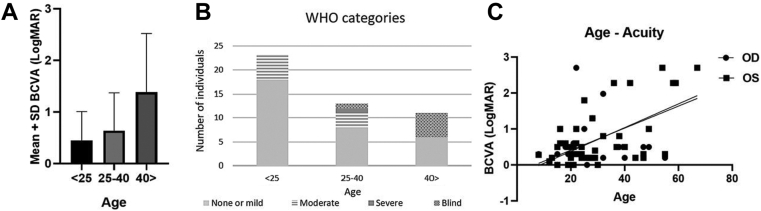
Visual acuity in *CERKL*-associated retinal dystrophy. **A,** Mean visual acuity ± standard deviation (SD) in patients from our cohort at their baseline visit, divided into 3 age groups. **B,** World Health Organization (WHO) visual impairment categories in the same age groups at their baseline visit. **C,** Linear regression showing a significant association between age and best-corrected visual acuity (BCVA) at baseline visit, for both right eye (OD) (*P* = 0.0002) and left eye (OS) (*P* < 0.0001).

Mean baseline BCVA was 0.7 ± 0.8 logMAR for the right eye (OD; median, 0.3) and 0.8 ± 0.8 for the left eye (OS; median, 0.4). In those aged 15–24 years (23 participants), BCVA ranged from 0.0 logMAR to light perception (LP, mean 0.5 ± 0.6 logMAR; median, 0.3; Fig 1 A, B), 18 had no or mild visual impairment, and 5 had moderate visual impairment. There were 13 patients aged 25–40 years old, whose BCVA ranged from 0.0 logMAR to hand movements (mean, 0.7 ± 0.7; median, 0.4): 8 had no or mild visual impairment, 3 had moderate visual impairment, 1 had severe visual impairment, and 1 was blind. Among those over 40 years old (11 individuals), BCVA was between 0.2 logMAR and LP (mean, 1.4 ± 1.1; median, 0.8): 6 had no or mild impairment, and 5 were blind. Asymmetric BCVA was seen in 10 patients (21%; 4 in the 15–24-year-old group, 5 in the 25–40-year-old group, and 1 in the 41-and-older subgroup). Analyzed cross-sectionally, there was a significant association between age and BCVA for both OD (*P* = 0.0002) and OS (*P* < 0.0001, Fig 1C).

Refractive data were available for 23 patients (49%), with a mean spherical equivalent of −1.9 ± 2.8 diopters (D). Of these, 17 patients (74%) had a mild-myopic or hyperopic spherical equivalent (−3 to +3 D), and the remaining 6 had moderate-to-high myopia.

### Clinical Examination, Color Fundus Photography, and Fundus Autofluorescence Imaging

Corneal examination was unremarkable in all cases. Eleven individuals (23%) developed lens opacities, at a mean age of 46.3 ± 15.2 (range, 26–73 years). There was no evidence of high intraocular pressure or glaucoma in any patient.

Ultrawide-field pseudocolor fundus and autofluorescence (AF) imaging was available in 39 participants (82%). The most common retinal feature was the presence of well-demarcated areas of atrophy at the macula, present in 27 patients (57%), from as early as 16 years of age. Fine hyperautofluorescent dots at the macula and perimacula (not visible on examination or color) were also seen in 23 patients (49%; age range, 6–60 years), and peripheral punched-out areas of chorioretinal atrophy were present in 21 patients (45%; Table 1) from 23 years of age, resembling a more extensive choroideremia-like pattern in patients with advanced disease.

**Table 1:** Clinical Characteristics of Patients with *CERKL*-Associated Retinal Dystrophy

Characteristics	Patients (n = 47)
Families	37
Gender, n (%)	
Female	22 (47)
Male	25 (53)
Age at first examination (mean ± SD, yrs)	29.6 ± 13.9
Age at last examination (mean ± SD, yrs)	39.2 ± 16.6
Follow-up time (mean ± SD, yrs)	9.1 ± 7.4
Ethnicity, n (%)	34 (72)
Asian	19 (56)
White European	13 (38)
African descent	2 (6)
Age of onset (mean ± SD, yrs)	19.8 ± 9 yrs
Reported first symptom, n (%)	
Central vision loss	19 (40)
Concurrent central vision loss and nyctalopia	16 (34)
Night blindness	5 (11)
Others (scotomas, photophobia, color vision issues)	7 (15)
Spherical equivalent refractive error (mean ± SD, D)	-1.9 ± 2.8
Funduscopic examination, n (%)	
Circumscribed areas of atrophy in the macula	27 (57)
Fine white dots at the macula and perimacula	23 (49)
Peripheral punched-out-like areas of chorioretinal atrophy	21 (45)
Hyperautofluorescent ring at the posterior pole	11 (23)
Ring including the optic disc	5 (11)
Hypoautofluorescent nummular dots that followed the vascular arcades	5 (11)
Mottled/uneven retinal aspect	3 (6)
Foveal-sparing maculopathy	3 (6)
Abnormal autofluorescence pattern	4 (9)
Retinal pigment deposits, n (%)	42 (89)
None	18 (38)
Minimal	15 (32)
Moderate	3 (6)
Dense, diffuse	6 (13)
Most affected retinal area by UWF imaging, n (%)	39 (82)
Macula	11 (23)
Periphery	11 (23)
Similar both	17 (36)
Full-field electroretinography, n (%)	30 (64)
Similar cone and rod involvement	16 (53)
Rod-cone pattern	8 (27)
Cone-rod pattern	3 (10)
Macular dystrophy (normal full-field ERG)	3 (10)

Abbreviations: D = diopters; ERG: electroretinogram; SD = standard deviation; UWF = ultrawide-field.

**Figure 2:**
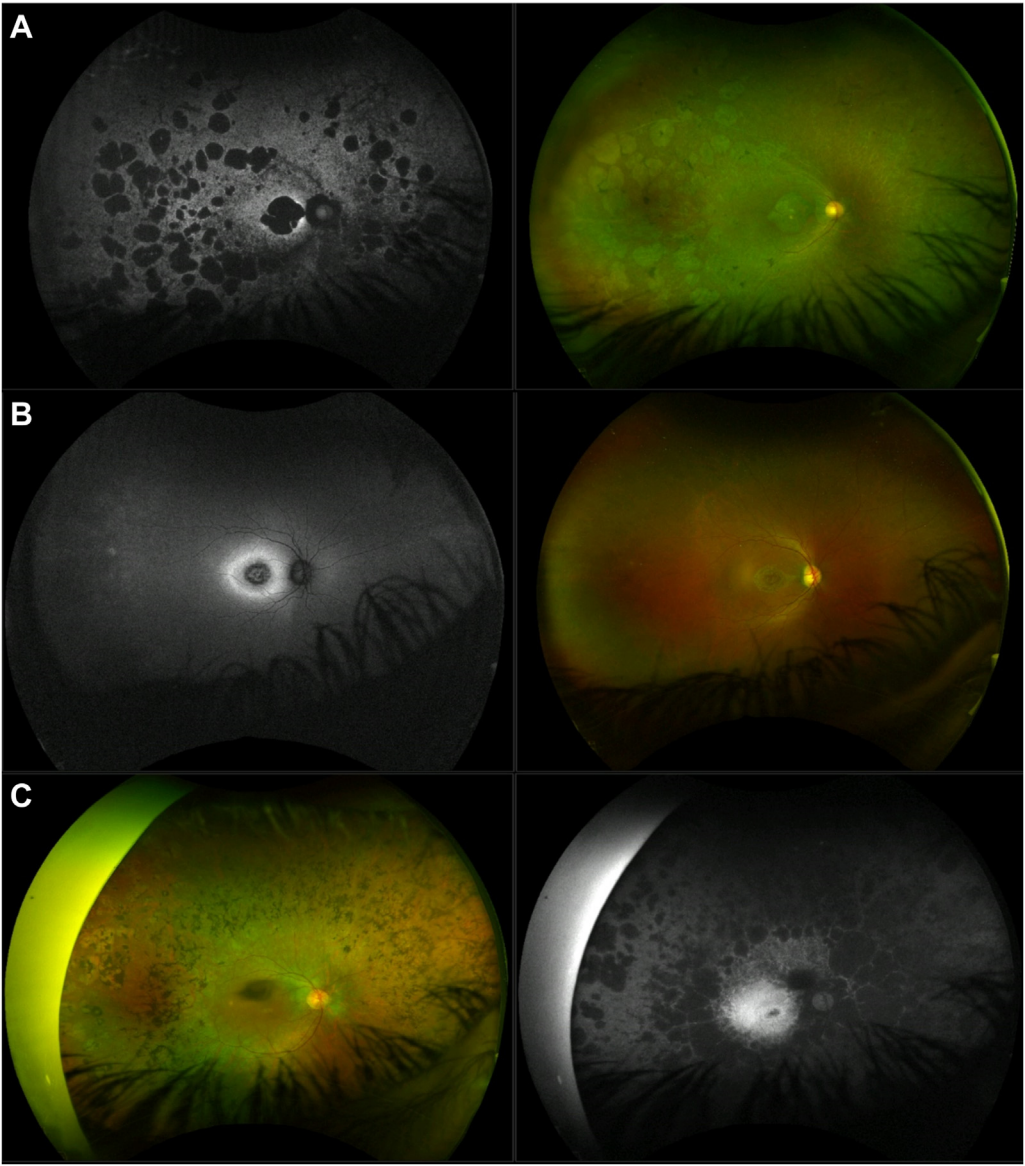
Fundus features of patients with *CERKL*-associated retinal dystrophy. **A,** 39-year-old patient with both macular and peripheral circumscribed patches of chorioretinal atrophy (CRA) and moderate pigment deposits. The patient had a rod-cone dystrophy on electrophysiological testing (electroretinogram [ERG]; ID, 10) and reported decreased central vision as her initial symptom, at age 15. **B,** A 21-year-old showing only macular atrophy, a hyperautofluorescent ring at the posterior pole, and no pigmentary changes. He had a cone-rod dystrophy on ERG (ID, 27) and described decreased central vision and light sensitivity since age 17. **C,** Retinal images from a 45-year-old patient with predominantly peripheral involvement, with CRA patches in the periphery and mid-periphery and diffuse pigment dispersion. She did not have an ERG assessment and mentioned concurrent decreased central vision and nyctalopia since age 36.

Eighteen patients did not have pigment deposits (38%; age range, 6–64 years), 15 had minimal bone-spicule-like (BSL) or nummular pigment (32%; age range, 23–72 years), 3 had moderate BSL deposits (6%; age range, 32–42 years), and 6 had dense, diffuse BSL pigment (13%; age range, 34–79 years). By assessing their imaging, 11 patients (23%) had predominantly macular involvement, 11 (23%) had prevailing peripheral involvement, and 17 (36%) had widespread retinal dystrophy affecting the posterior pole and the peripheral retina similarly (Fig 2; Table S2, available at www. ophthalmologyretina.org).

**Figure 3:**
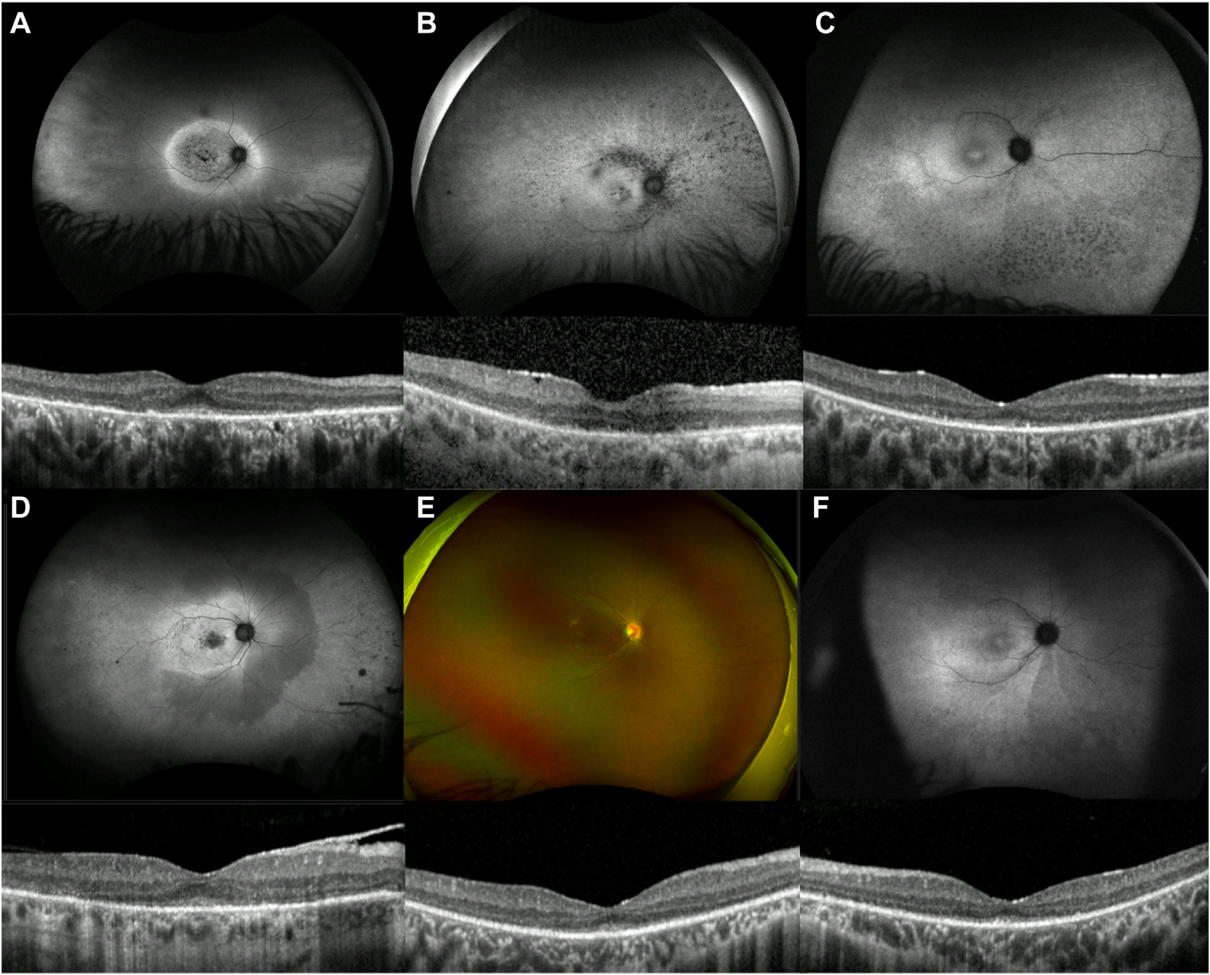
Less-common retinal features found in our *CERKL*-associated retinal dystrophy cohort. **A,** Autofluorescence (AF) imaging from a 30-year-old man, with a hyperautofluorescent ring at the posterior pole, extending beyond the temporal vascular arcades and encompassing the optic disc. Macular OCT scan showing remnant subfoveal ellipsoid zone (EZ) line. The patient had equally affected rod and cone electroretinogram (ERG) responses (ID, 22) and was diagnosed with a “macular dystrophy” at age 19. **B,** Thirty-two-year-old patient with a hypoautofluorescent pattern that follows the vascular arcades. Macular OCT scan is positive for outer retinal degeneration and an epiretinal membrane (ERM). She had equal rod and cone dysfunction on ERG (ID 3) and reported decreased central vision as her initial symptom, at age 7. **C,** AF imaging from a 15-year-old patient with uneven autofluorescence and hypoautofluorescent dots inferiorly. Macular OCT shows minimal subfoveal EZ and outer retinal degeneration. He had equal rod and cone impairment on ERG (ID, 13) and developed decreased central vision at age 13. **D,** A 55-year-old patient with a few peripheral CRA patches and an abnormal autofluorescence pattern, with a preserved nasal, mid-peripheral area. Macular OCT shows minimal subfoveal EZ, outer retinal degeneration, and ERM. She had a rod-cone dystrophy pattern on ERG (ID 24) and described decreased central vision since age 23. **E,** Nine-year-old (ID 41, brother of ID 13 displayed on C). There is vessel thinning, mild retinal mottling, and uneven autofluorescence. Bilateral macular OCT is positive for outer retinal loss, with preserved central EZ and overall architecture. He remains asymptomatic (last visit at age 11).

Common IRD signs were also present in this cohort, such as peripapillary atrophy (seen in 35 patients, 74%) and a hyperautofluorescent ring at the posterior pole, appearing either entirely inside the temporal vascular arcades (11 patients) or also encompassing the optic disc (5 individuals; Fig 3A). Other less frequent features were hypoautofluorescent nummular dots that followed the vascular arcades (5 patients; Fig 3B), a mottled retinal AF pattern (3 young patients; Fig 3C), foveal-sparing maculopathy (3 patients), and abnormal AF patterns, with preserved mid-peripheral retina (4 patients; Fig 3D and E).

### Macular OCT Analysis

Forty-one individuals had macular OCT scans, Heidelberg blue autofluorescence (BAF), and infrared (IR) imaging (87%).

Baseline central macular thickness (CMT) was 180.2 ± 36.0 μm (mean age, 30.2 ± 13.8 years; range, 7–71 years). Outer nuclear layer thickness was quantifiable in 33 patients (66 eyes [80%]; range, 7–51 years), with a mean value of 38.7 ± 24.4 μm. Both CMT and ONLT were significantly lower than the unaffected population (*P* < 0.001). Foveal EZ was present in 28 patients (52 eyes; range, 7–51 years) with a mean width of 350.5 ± 302.9 μm. A subfoveal hyporeflective zone was seen in 4 eyes (3 patients; range, 31–50 years). Considering cross-sectional data only, a significant association was observed between older age and narrower EZ width (*P* = 0.05), and no significant association was found between age and CMT (*P* = 0.08) or ONLT (*P* = 0.35). No significant association was found between BCVA and EZ, CMT, or ONLT (*P* = 0.22–0.65).

**Figure 4:**
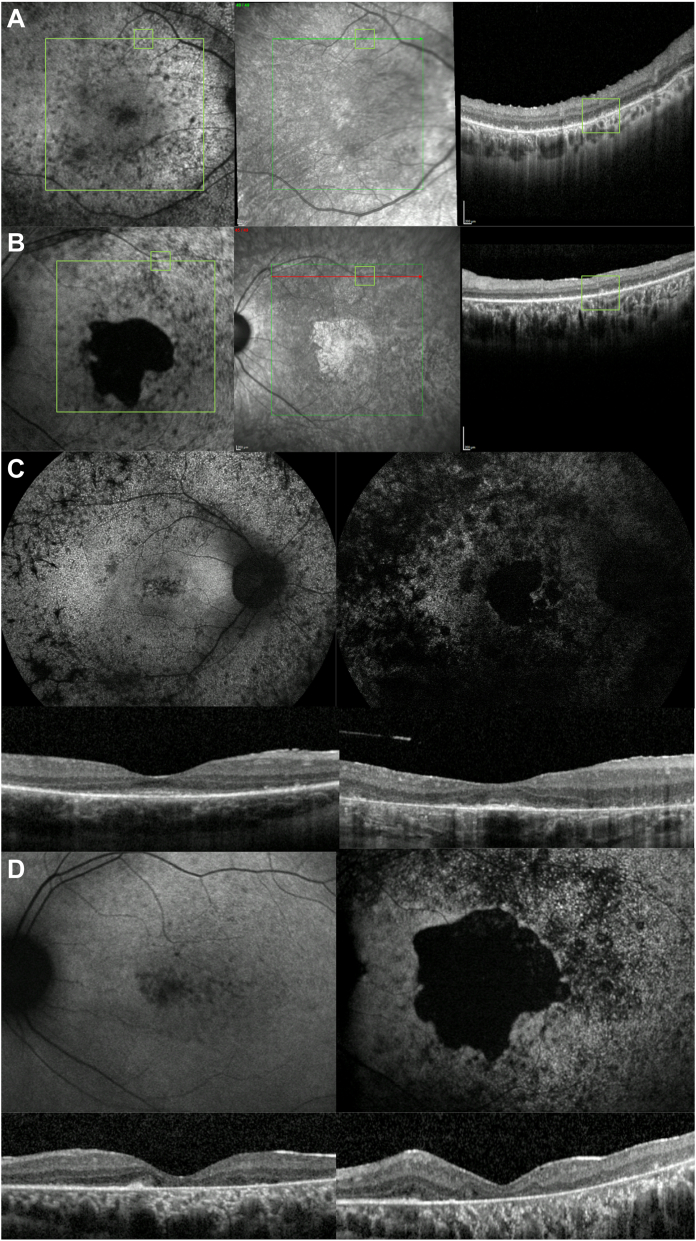
Macular OCT and Heidelberg imaging. **A,** Correlation of blue fundus autofluorescence (BAF), infrared (IR) and OCT imaging of patient ID 14 at 20 years of age, with rod-cone dystrophy on electroretinogram and mixed rod and cone symptoms at onset. A hyperautofluorescent dot is seen in BAF next to the vessel (green square), hyporeflective in IR and subretinal hyperreflective material evident in that location on OCT. **B,** Correlation of BAF, IR and OCT imaging of patient ID 20 at age 27. Here is another example of a hyperautofluorescent dot on BAF, hyporeflective on IR and subretinal hyperreflective material in that location. **C,** Fundus autofluorescence images of a patient clinically diagnosed with *CERKL*-associated retinitis pigmentosa (ID 43). To the left is the patient at 26 years of age, and to the right, that same patient at age 37. Scattered atrophy is seen in the posterior pole and mid-periphery, with hyperautofluorescent fine dots surrounding the posterior pole at an early stage, and few dots remaining in the most recent image, these having been largely replaced by hypoautofluorescence. Macular OCT scans show a narrowed EZ line and loss of retinal layering over follow-up. **D,** Fundus autofluorescence changes in a patient (ID, 20) with an electrophysiological diagnosis of cone-rod dystrophy and mixed rod and cone symptoms at onset. To the left is the posterior pole at age 18 and to the right, at age 33. In this case, the central atrophy enlarged, and the hyperautofluorescent dots increased in number/became more noticeable over time. Longitudinal macular OCT shows more profound outer retinal atrophy and poor retinal architecture over time.

Subretinal hyperreflective material was the most common finding, present in 36 individuals (88%; Fig 4), and this was often correlated with fine hyperautofluorescent dots detected with BAF. These dots were noticed in the posterior pole or surrounding it in 29 patients (71%) and were not discernible with IR or color imaging. Other findings were hyperreflective material in the inner retina (33 individuals [80%]), epiretinal membrane (ERM; 27 patients [66%]), and cystoid macular edema, present in 3 patients (2 with RP and 1 with CORD).

### Electrophysiological Assessment

**Figure 5:**
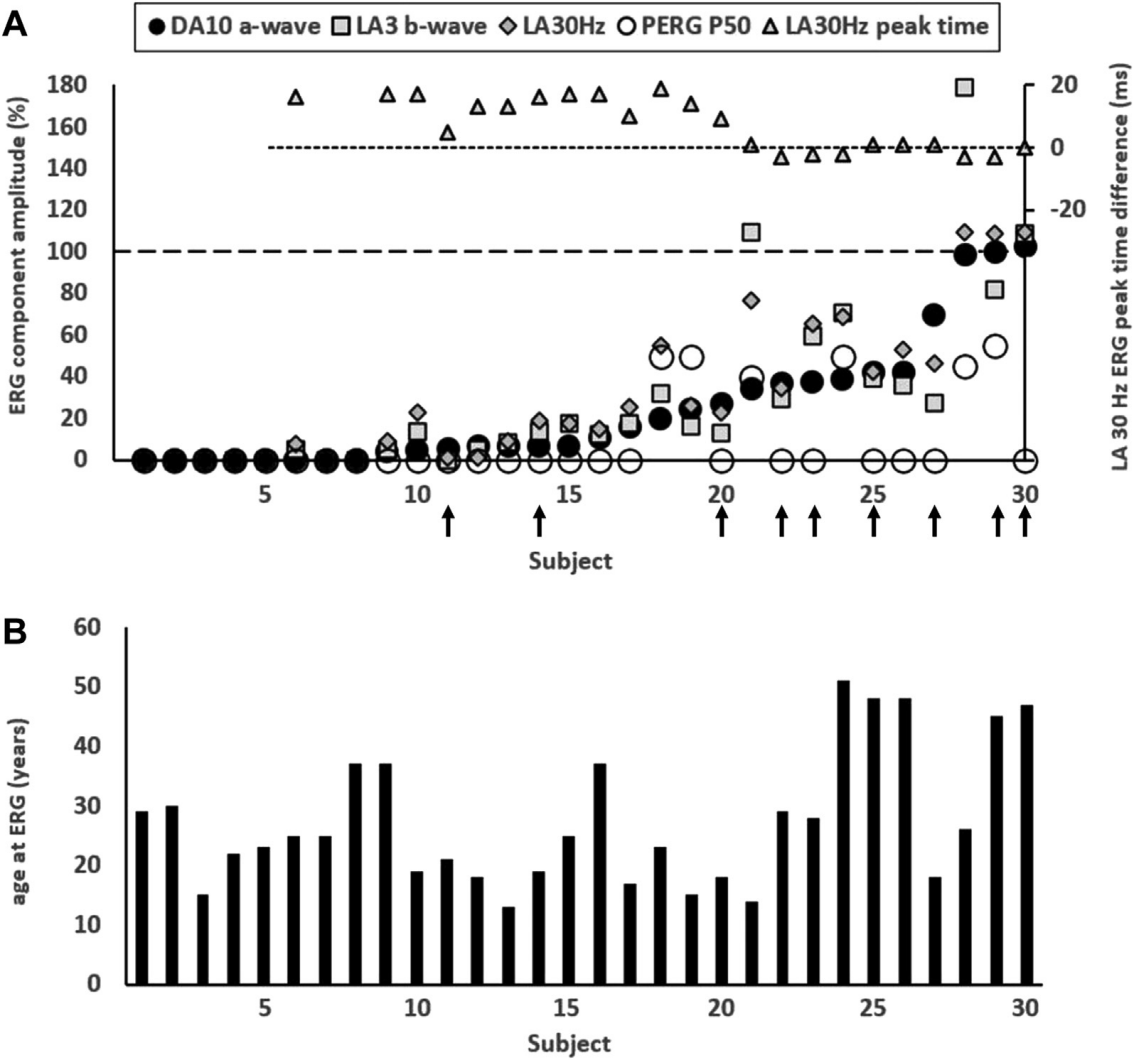
Full-field electroretinogram (ERG) and pattern electroretinogram (PERG) findings summarized in 30 patients tested according to the International Society for Clinical Electrophysiology of Vision standard. **A,** The amplitudes of the dark-adapted (DA) 10 ERG a-wave, LA 30 Hz ERG, LA 3 ERG b-wave, and PERG P50 component are plotted against the primary axis as a percentage of the age-matched lower limit of the reference range (horizontal broken line), with values arranged in ascending order of DA 10 ERG a-wave amplitude for clarity. The light-adapted (LA) 30 Hz peak times are plotted against the secondary axis as a difference from the age-matched upper limit of the reference range (horizontal dotted line). **B,** The age of the patients at the time of testing, arranged in same order as in (**A**). Patients with non–double-null variants include those numbered 11, 14, 20, 22, 23, 25, 27, 29, and 30 (highlighted with vertical arrows). All DA and LA ERGs and PERGs were undetectable in patients 1–5, 6, and 7.

Thirty patients had electrophysiological assessment (64%; Tables 1 and S2). Sixteen of them showed a similar degree of DA and LA ERG reduction, in keeping with a moderate-to-severe rod and cone photoreceptor dystrophy, including 7 (age range, 15–30 years) with undetectable DA and LA ERGs. The ERG findings in others revealed a rod-cone (N = 8; age range, 14–51 years; median, 24 years) or cone-rod (N = 3; age range, 15–28 years) pattern of dysfunction, with a further 3 showing normal or near-normal ERG amplitudes (cases 28–30; ages 26, 45, and 47 years; Fig 5). The LA 30Hz ERG peak times were normal or borderline (= 95th percentile) in the 3 cases with preserved ERG amplitudes (MD group). Light-adapted 30Hz ERG peak times were worse in the 13 patients with the smallest detectable ERGs (median delay, 13 ms) and were normal or borderline in the remaining 10, including the 5 oldest patients (range, 45–51 years).

**Figure 6:**
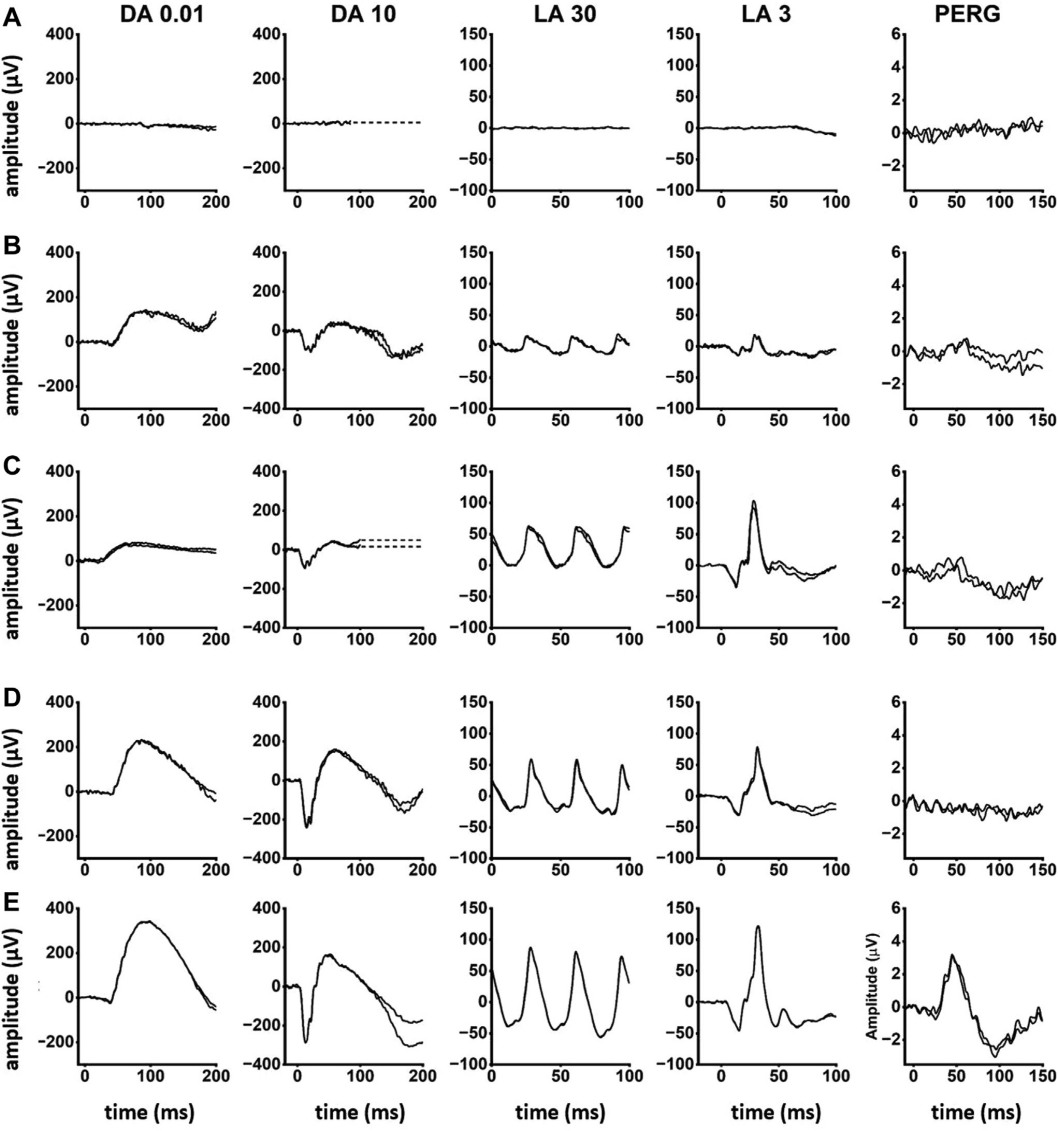
Representative full-field and pattern electroretinograms (ERGs) from 3 patients and 1 control subject for comparison. **A,** patient 2 (aged 30 years); **B,** patient 22 (29 years); **C,** patient 30 (47 years); **D,** representative control (“normal”) recordings. Numbering of patients corresponds to that used in Figure 5. Data are shown for the right eyes only, as all recordings showed a high degree of interocular symmetry. Patient traces are superimposed to demonstrate reproducibility. Broken lines replace blink artefacts for clarity. The full-field ERGs in patients 2 and 22 are consistent with a rod and cone photoreceptor dystrophy affecting rods and cones similarly, with pattern electroretinogram (PERG) evidence of macular involvement, more severe in patient 2. The ERGs in patient 21 are in keeping with a rod-cone dystrophy, with a detectable but subnormal PERG, indicating macular involvement. The undetectable PERG in case 30 is consistent with severe macular dysfunction with no full-field ERG evidence of generalized retinal dysfunction.

Comparison of ISCEV-standard DA 0.01, DA 3, and DA 10 ERG a- and b-waves, LA 30Hz ERG and LA 3 (single flash) ERG b-waves revealed a high degree of interocular symmetry in amplitudes (slope = 1.03; *r*^*2*^ = 0.98) and peak times (slope = 1.02; *r*^*2*^ = 0.99). Figure 5 summarizes the electrophysiological findings (right eyes) and patient ages at the time of testing, and Figure 6 shows representative recordings.

There was no significant correlation between age and the amplitudes of the DA 0.01 ERG, DA 10 ERG a- and b-waves, LA 30Hz ERG or LA 3 ERG b-waves, or the peak times of the LA 30Hz ERG. Pattern ERG P50 was undetectable in 24 patients in keeping with severe macular dysfunction. Of the remaining 6 cases, including 2 with MD, the PERG P50 component was reduced by 45%–60% (Fig 5A). Flash ERGs in the 5-year old child tested with skin electrodes showed only residual photopic ERGs, markedly subnormal scotopic bright flash ERGs, and an undetectable PERG P50 component (data not shown), consistent with a photoreceptor dystrophy with severe macular involvement.

Follow-up ERG data were available in 3 patients (Fig S7, available at www.ophthalmologyretina.org), showing rod-cone (ID, 21) or cone-rod (IDs, 19 and 20) patterns of dysfunction at baseline. In patient 21, there was marked reduction in all detectable ERG components and a 15-ms increase in LA30Hz ERG peak time over a 13-year period (from age 14–27 years). In patient 19, there was rapid reduction in DA ERG amplitudes with no significant change in LA ERG amplitudes or peak times (from age 15–16 years). In patient 20, there was significant reduction in all ERG components with borderline (2 ms) worsening in LA 30Hz ERG peak time (from age 18–23 years).

### Longitudinal Analysis

Mean follow-up time was 9.1 ± 7.4 years (range, 0–21 years). Follow-up BCVA was available in 34 (72%) patients and was 1.5 ± 1 logMAR OD (median, 1.65) and 1.65 ± 1.05 OS (median, 2.28; mean age, 42 ± 14 years). The rate of BCVA decline was 0.08 logMAR (4 letters)/year, and there was a significant difference between baseline and follow-up BCVA (*P* < 0.0001).

During the first 5 years of follow up (n = 34), the rate of BCVA decline was 0.08 logMAR (4 letters)/year for both the overall cohort and for the group aged 15–24 years old, 0.13 (6.5 letters)/year for the 25–40-year-old group and 0.04 (2 letters)/year for the 41 and older subgroup. During years 5–10 of follow up (n = 23), the rate was 0.09 logMAR (4.5 letters)/year for the 25–40-year-old group and 0.05 (2.5 letters)/year for the older subgroup. Lastly, during the period of 10–15 years of follow up (n = 12), the group aged 25–40 years old had a decrease rate of 0.08 logMAR (4 letters)/year and the older subgroup of 0.06 (3 letters)/year.

Twenty-five patients out of the 34 with longitudinal data (74%) had a decrease in BCVA of 15 ETDRS letters or more over follow-up in at least 1 eye, 20 (59%) during the first 5 years since their baseline visit. Seventeen patients (50%) progressed to more advanced WHO categories of visual impairment over follow up, 11 (32%) of whom became blind.

Thirty-three individuals had longitudinal macular OCT scans during a period of up to 12 years (mean of 6.4 ± 3.5). Longitudinal analysis demonstrated significant differences between baseline and follow-up CMT (*P* < 0.0001, −5.9 μm/year), ONLT (*P* < 0.0001, −2.7 μm/year), and EZ width (*P* < 0.0001, −34.5 μm/year). Two thirds of the patients had decreased CMT over follow-up, and one third had increased values. The ONL was noticed to be isointense to contiguous layers, less distinct, and unable to be quantified in 9 patients over the follow-up period. The association between BCVA and EZ, CMT and ONLT remained not significant (*P* = 0.16 to 0.57). Fifteen out of 16 patients with a hyperautofluorescent ring at the posterior pole had a follow-up assessment. In 9 patients, the ring grew in diameter; in 5, it became smaller; and in 1, it faded.

### Molecular Genetics

**Figure 8:**
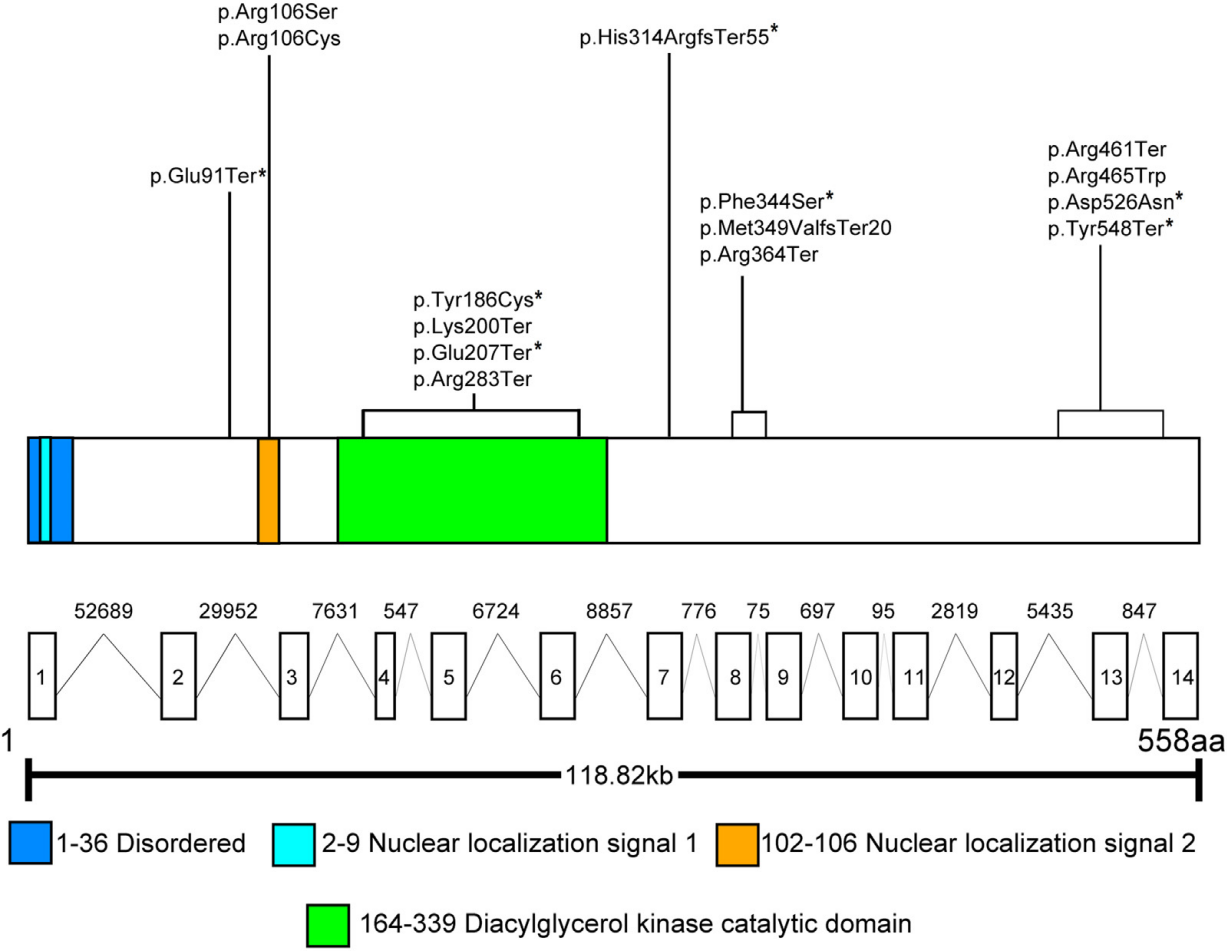
Graphic representation of the *CERKL* gene and protein, with details on functional domains. *CERKL* (Transcript; NM_001030311.2, ENST00000339098.9, Uniprot accessions: Q49MI3) contains 13 exons that encode a 532-amino-acid protein containing a disordered region, nuclear localization signal 1 motif, nuclear localization signal 2 motif, and a diacylglycerol kinase catalytic domain. Seven nonsense, 6 missense, and 2 small-deletion/frameshift variants detected in this cohort are demonstrated. ∗indicates novel variants.

All patients had biallelic/multiple rare variants in *CERKL*, and their molecular characteristics are listed in Table S3 (available at www.ophthalmologyretina.org). Thirty-two patients had homozygous variants. Twenty-three different variants were present in our cohort: 7 nonsense, 6 splice-site alteration (3 canonical splice site, 2 intronic deletions, and 1 intronic change), 6 missense, 2 small deletions/frameshift, and 2 large deletions. Eight were classified as pathogenic, 8 as likely pathogenic, and 8 as VUS. Nine (39%) were novel variants, and 14 were previously reported elsewhere. Schematic representation of these detected variants and the status of evolutionary conservation are presented (Fig 8; Fig S9, available at www.ophthalmologyretina.org).

The most common variant was p.(Arg283Ter), encountered in 17 homozygous patients and in 6 presumed compound heterozygotes. The second most frequent variant was p.(Arg106Ser), present in 4 homozygotes and 2 compound heterozygotes. Thirty-six participants (77%) had DN genotypes (nonsense, frameshift, splicing, or exon deletion), 4 were compound heterozygous of a null and a missense variant, and 7 had 2 missense changes (11 NDN; Table S3, available at www. ophthalmologyretina.org). One patient (ID, 30) was homozygous for a VUS and had a phenotype that matched *CERKL*-retinopathy; hence, he was included in the cohort and considered as a likely *CERKL*-associated case.

### Genotype-Phenotype Correlation Analysis

Fifteen out of the 16 patients with childhood-onset symptoms had DN genotypes. The mean age of onset in individuals with NDN variants was 22.8 ± 9.3 years (median, 18.5 years) and 19.5 ± 8.7 for DN (median, 19 years; *P* = 0.36). At baseline, the 5 patients aged 15–24 years with moderate visual impairment harbored DN variants; the 5 patients aged 25–40 years with moderate visual impairment or worse had DN variants; and among those over 40 years old, 4 out of 5 that were blind also had DN variants. The rate of VA decline was 0.07 ± 0.07 logMAR/year in the NDN group and 0.08 ± 0.08 logMAR/year in the DN group. There were no significant differences between DN and NDN groups regarding rate of progression, and no specific variant was found to be consistently milder than the others or associated with a particular phenotype.

Regarding fundus features, 8 out of 11 (73%) NDN patients had no pigment deposits, and the remaining 3 had minimal BSL pigment (age range, 20–72 years). In contrast, among 25% of patients with DN genotypes (n = 28 with UWF imaging), 8 had no pigment deposits (29%; age range, 14–64 years), 10 had minimal pigment, 3 had moderate pigment, and 4 had dense/diffuse pigmentary changes (age range, 34–79 years). The presence of a ring of increased AF at the posterior pole was noted in 50% of DN patients (14/28) and 27% of NDN (3/11).

Concomitant peripheral and macular circumscribed areas of atrophy were observed in 4 patients with NDN genotypes versus in 15 with DN genotypes. The DN subgroup included 6 patients with more marked central retinal involvement, 13 with similar central and peripheral involvement, and 9 with the peripheral retina mostly affected. The NDN group consisted of 5 patients who had a predominantly macular involvement, 4 who had similar central and peripheral involvement, and 2 who had a more peripheral degeneration (Table S2).

The ERG abnormalities were most severe (DA10 ERG a-wave reduction) in 18 of 21 with a DN genotype, including 7 with undetectable ERGs. The 9 individuals harboring NDN variants included 3 of the 5 oldest subjects tested, with 3 of the 5 mildest ERG phenotypes (2 with MD; Fig 5). Of the 10 patients with normal or borderline LA30Hz ERG peak times, 6 harbored NDN variants.

There was significant concordance of phenotype within families, with 7 sets of siblings displaying similar phenotypes, including degree of pigment deposits and atrophic patches, and a similar age of onset (± 5 years). Interestingly, 3 young siblings were diagnosed with RP and had a comparable presentation, whereas their mother had a CORD phenotype (IDs 8, 13, 40, and 41). No correlations were found regarding variants’ location or gene domain and phenotype.

## Discussion

This study examines the detailed clinical and functional phenotype in the largest cohort of genetically characterized patients with *CERKL*-associated retinopathy to date. The description of the uncommon MD presentation is extended. Nine novel disease-causing variants are identified, and genotype-phenotype correlations examined. Comprehensive retinal imaging, quantitative electrophysiological and natural history data are detailed, aimed at facilitating the diagnosis and establishing rates of disease progression, to inform future patient management.

*CERKL*-retinopathy has been categorized to date as either presenting as RCD or CORD, even in patients with the same genotype, excluding possible genotype-phenotype correlations.^15^^,^^17^^,^^25^ It is of note that detailed review has demonstrated that overlapping features of both categories were present concurrently in a large proportion of patients, with an early drop in acuity and central visual field loss in patients with “RCD,” and nyctalopia and peripheral visual field loss as presenting symptoms in individuals with “CORD” (Table S4, available at www.ophthalmologyretina.org).^15^^,^^17^^,^^25^ It appears that unlike the majority of other genetic causes of IRD, the boundaries between the 2 categories are not as clear, with many patients presenting with both cone- and rod-related features simultaneously (in our cohort, 36% on imaging, 34% on ERG, and 34% as per initial symptoms). This has been noted in previous reports of smaller cohorts, but it is extended and confirmed in our larger, genetically and ethnically diverse cohort (Table S2, available at www.ophthalmologyretina.org).^5^^,^^17^

The rate of VA decline was high compared with other genotypes (*CRB1*-RP 0.07 logMAR/year and *RPGR*(ORF15) 0.02/year)^24^^,^^26^ and even faster in the 25–40-year-old group, suggesting preserved acuity until the third decade of life, followed by a more rapid decline. Yet VA encompassed an incredibly broad range both at baseline and during follow-up, with over 50% of individuals above 40 years old still having no or mild visual impairment as per the WHO classification, indicating the variable prognosis associated with *CERKL*-retinal dystrophy. Asymmetric BCVA was seen in 21% of patients at baseline, which is high compared with other conditions including Stargardt disease (reported at 8%).^27^ If a less stringent measure of VA asymmetry was applied, an even greater asymmetry would be noted.

Macular hyperautofluorescent punctate lesions visible with BAF were found to correspond with subretinal hyperreflective material on OCT (Fig 4A, B). This feature was also highlighted by Sengillo et al,^28^ who described them as becoming denser as the disease progressed. In our cohort, we observed 2 scenarios over time: 1 in which the hyperautofluorescent lesions were replaced by hypoautofluorescence and increased atrophy, and another where indeed these dots increased in number as the atrophy grew (Fig 4C, D). The fact that these lesions are excited by 488-nm blue light could indicate that they are at least partly composed by lipofuscin/lipofuscin-like and N-retinylidene-N-retinylethanolamine (A2E)/A2E-related material, suggesting that they may correspond to photoreceptor debris resultant from *CERKL*-mediated decreased retinal pigment epithelium (RPE) phagocytosis.^29^^,^^30^ Similar dots/subretinal debris-material has been reported in *MERTK*-retinopathy,^31^ a dysfunctional phagocytosis-associated dystrophy.

A novel, less-common finding in our cohort is the presence of a large hyperautofluorescent ring extending beyond the posterior pole and encompassing the optic disc (5 patients; Fig 3A; Table S2, available at www.ophthalmologyretina.org). This pattern has also been seen in other IRDs including dominant *NR2E3*- and *EYS*-related retinopathies, representing in both cases the boundaries between affected and unaffected retina.^32^^,^^33^ Yet in *NR2E3*, this ring grew centrifugally toward the mid-periphery, and in *EYS,* it shrunk over time. In our case, the ring grew and became less well-defined in the majority of the patients, in keeping with *NR2E3-*IRD. Other features to consider in *CERKL*-retinopathy are peripapillary atrophy, which, although not pathognomonic of *CERKL*, may be useful when distinguishing between this gene and *ABCA4* (classically associated with peripapillary sparing)^34^ and ERM with inner retinal wrinkling. The latter was reported in up to 22.8% of patients with RP; however, in our cohort, it was seen in nearly 3 times that proportion, and in previous series, it affected 2 and 3 out of 6 patients.^15^^,^^35^^,^^36^

The electrophysiological profiles in our series were diverse, with 16/30 having a similar degree of rod and cone photoreceptor dysfunction and most others (8/30) having a rod-cone pattern of dysfunction. A minority had CORD and MD (3/30 each), in keeping with previous reports of ERG variability.^5^^,^^10^^,^^15^ There was an overlap in ERG phenotypes associated with DN and NDN genotypes, but a large majority of nullizygous cases had severe generalized photoreceptor dysfunction, including the youngest individuals. Those with NDN variants included 2 older patients with MD (normal ERG) and most of those with relatively mild retinal dysfunction. An apparent disconnect between ERG and initial symptoms was seen in 4 patients with RCD on electrophysiology and initial symptoms of opposite phenotypes (CORD and MD; ID 6A, 10C, 23, and 24), all with equal central and peripheral involvement on imaging. Although in some of these cases, the symptoms may evolve to eventually match the ERG phenotype (ERG functioning as a predictor, as in *ABCA4*-Stargardt disease),^37^ in others, the ERG may represent an additional piece of information that characterizes the patients' disease, not necessarily matching the other assessments. The lack of clear correlation between the ERG findings, symptoms, and retinal imaging (Table S2, available at www.ophthalmologyretina.org) highlights the need for comprehensive phenotypic assessment.

In our study, p.(Arg283Ter) was the most common variant, in concordance with other reports.^17^ The second most common change, p.(Arg106Ser), was described to possibly lead to cellular death given its highly conserved location within the protein, functioning as a null allele despite being missense.^38^ We have demonstrated a degree of genotype-phenotype correlation, with patients harboring DN genotypes displaying signs of increased severity when compared with those with NDN, including younger age of onset, greater visual impairment, more retinal pigmentary disturbance with often concomitant peripheral and macular circumscribed atrophic lesions, and more profound ERG dysfunction. However, we also noted overlap between the 2 genotypic groups and the aforementioned signs of disease severity (in keeping with the previously well-documented heterogeneity of IRDs). Investigation of further *CERKL* cases will help to extend our observations.

Over 20 different *CERKL* transcripts have been found in the human retina due to extensive alternative splicing and multiple translational start sites, with preferential isoform expression in different cells.^39^^,^^40^ A double-knockout zebrafish model found that rods degenerated earlier and more significantly than cones,^30^ yet a knockdown-knockout mouse model found cones to be more affected.^40^ The models agreed on photoreceptor outer segment abnormalities, accumulating in the interphotoreceptor matrix and becoming very long and disorganized, suggestive of decreased RPE phagocytosis.^30^^,^^40^ *CERKL* has recently been characterized as an oxidative stress-protective gene within RPE cells; hence, decreased RPE function may occur in its abscence.^14^ It is possible that different disease-causing variants have greater impact on certain transcripts preferentially expressed in cones versus rods, or equally in both, resulting in the different phenotypes and symptoms observed in our study. Furthermore, certain variants could affect how CERKL interacts with other proteins, interfering with its complex function and regulation, potentially again with different impact on rods versus cones. Environmental or other genetic modifying factors may also play a role in the variable phenotypic presentations. Future functional studies may shed further light on this topic.

Given the link between *CERKL* and oxidative stress, antioxidant therapeutic approaches might have a positive effect on this condition.^41^ These are currently under investigation for other IRD genes such as *ABCA4* and if successful, might also prove useful for *CERKL* retinopathy.^42^^,^^43^ Similarly, slowing down the visual cycle could decrease the generation of A2E and limit the formation of macular hyperautofluorescent punctate lesions. RBP4-inhibitors could be helpful if proven beneficial in *ABCA4* Stargardt disease (NCT05244304). *CERKL* would also be a good candidate for gene therapy, given its reasonable window of opportunity. However, due to its size not fitting in a regular AAV-vector, it would require alternative vectors or dual vector technology.

Our study limitations include its retrospective nature, the different proportion of individuals in the DN and NDN subgroups, not all data being available for every individual, and data being acquired with various methods and protocols. The lack of visual field data reflects the clinical environment in which these data were collected. These are, to a large extent, offset by the large number of genetically confirmed patients, their wide age range and ethnic background, and the international nature of the cohort.

This study represents the largest series of patients with *CERKL*-associated retinopathy to date. We characterized detailed clinical features, disease progression, estimating the rate of VA change and providing insights into genotype-phenotype correlations. *CERKL*-retinopathy has a broad phenotypic spectrum ranging from isolated macular dystrophy to severe generalized retinal involvement, and it often does not follow the classical RCD/CORD dichotomy. In addition, it can present with both cone- and rod-related symptoms at the outset and may be associated with structural and functional signs of both cone and rod dysfunction/loss. In many patients, a greater rate of central vision loss may occur in the third decade. Common retinal changes observed in *CERKL*-retinopathy include peripheral and macular circular areas of chorioretinal atrophy, little to no pigment deposits, and fine hyperautofluorescent macular dots. The nature of retinal dysfunction often cannot be inferred reliably from clinical signs or imaging alone, highlighting the importance of comprehensive phenotyping.
