# Supplementary material for: CERKL-Associated Retinal Dystrophy: Genetics, Phenotype, and Natural History
Source: Ophthalmol Retina. 2023 Oct;7(10):918–31. doi: 10.1016/j.oret.2023.06.007 (PMC11108804; doi:10.1016/j.oret.2023.06.007)
Supplement: Supplementary Table 2 [file mmc1.pdf]

Supplementary Table 2. Clinical, electrophysiological, molecular, and imaging characteristics of the members of the cohort. Patients with the same letter next to their ID are from the same family.

ID	GC	Ethnicity	Sex	Age of onset (y)	Category of presenting symptoms	Initial exam			Final exam				Imaging					ERG group	Genotype		Segregation
						Age	RE (LogMAR)	LE (LogMAR)	FU time	Age	RE (LogMAR)	LE (LogMAR)	White dots	Primary involvement	Circular CRA	Macular hyperAF ring	Pigment				
1	42656	NA	m	24	RCD	29	0.2	0.2	2	31	0.2	0.2	Y	PERIPHERAL	P & M	N	Diffuse BSL	R=C	Hom	p.(Arg283Ter)	NA
2	43950	NA	f	28	MIXED	29	0.4	0.40	9	38	1.8	PL	Y	EQUAL	N	N	Minimal nummular/BSL	R=C	Hom	p.(Met349ValfsTer20)	NA
3	27801	Afghan	f	7	CORD	16	0.6	1	16	32	HM	HM	Y	PERIPHERAL	P & M	Y	Minimal	R=C	Hom	p.(Arg364Ter)	NA
4	40138	Asian Pakistani	m	24	MIXED	22	2.7	0.6	10	32	PL	PL	N	EQUAL	N	Y	None	R=C	Hom	p.(Glu207Ter)	NA
5	28600	Polish	f	12	MIXED	24	0.5	0.5	11	35	0.8	0.6	Y	PERIPHERAL	N	Y	Minimal BSL	R=C	p.(Arg461Ter)	Exon 2 deletion	In trans
6a	4970	White British	m	NA	CORD	28	1.3	1.3	3	31	1.5	1.8	N	NA	P & M	Y	Moderate BSL	RCD	p.(His288RfsTer55)	c.1238-1G>A	In trans
7	28522	Asian Indian - Hindu Gujerati	f	15	MIXED	24	0.3	0.30	18	42	HM	1.68	Y	PERIPHERAL	N	N	None	R=C	Hom	p.(Arg283Ter)	NA
8b	28726	Asian Pakistani	f	16	MIXED	36	2.28	2.28	11	47	HM	HM	Y	CENTRAL	P & M	N	Minimal BSL	R=C	Hom	p.(Arg283Ter)	NA
9	27882	NA	f	20	MD	32	0.2	0.80	19	51	PL	PL	Y	NA	P & M	Y (large)	None	R=C	Hom	p.(Met349ValfsTer20)	NA
10c	21928	Asian Pakistani	f	15	CORD	20	0.2	0.2	20	40	CF	CF	Y	EQUAL	P	N	Minimal	RCD	Hom	c.1151+3_1151+6delAAGT	NA
11	40308	Asian	m	16	MD	21	0.3	0.3	0	21	NA	NA	N	EQUAL	M	Y	None	R=C	p.(Arg283Ter)	p.(Arg106Ser)	In trans
12	41438	NA	m	19	MIXED	21	0.60	0.3	3	24	1	0.60	N	EQUAL	N	N	None	R=C	Hom	p.(Tyr548Ter)	NA
13b	46584	Asian Pakistani	m	13	MD	13	0.2	0.2	3	16	0.1	0	N	EQUAL	M	Y	None	R=C	Hom	p.(Arg283Ter)	NA
14	45356	NA	m	16	MIXED	19	0.3	0.3	2	21	0.3	0.3	N	PERIPHERAL	P & M	N	Dense BSL	RCD	p.(Arg283Ter)	p.(Tyr186Cys)	NA
15d	28063	White Irish	m	25	MIXED	25	1	1.80	17	42	HM	HM	N	EQUAL	N	Y (large)	None	RCD	Hom	p.(Arg283Ter)	NA
16	3607	Asian Indian	m	37	MIXED	40	0.2	0.50	21	61	PL	PL	N	EQUAL	M	Y	None	R=C	Hom	p.(Arg283Ter)	NA
17e	41050	Black African	m	13	MD	16	1	0.60	4	20	0.9	1	Y	NA	P & M	Y	Minimal BSL	R=C	p.(Arg461Ter)	c.613+2T>C	NA
18c	31090	Asian Pakistani	f	24	MIXED	24	0.2	0.2	12	36	HM	0.5	Y	EQUAL	P & M	N	Minimal	RCD	Hom	c.1151+3_1151+6delAAGT	NA
19	28558	NA	m	15	CORD	15	0	0.00	10	25	0.2	0.2	N	NA	M	Y	NA	CRD	c.238+2T>C	Exon 1 deletion	In trans
20f	26199	Asian Pakistani	m	18	MIXED	20	0.2	0.2	17	37	0.6	PL	Y	CENTRAL	N	N	Minimal BSL	CRD	Hom	p.(Arg106Ser)	NA
21a	4971	White British	m	20	MIXED	19	0	0	18	37	0.6	0.3	Y	PERIPHERAL	P & M	N	Minimal	RCD	p.(His288RfsTer55)	c.1238-1G>A	In trans
22f	43613	Asian Pakistani	m	19	MD	27	0.2	0.20	3	30	0.3	0.3	Y	CENTRAL	P	N	Minimal BSL	R=C	Hom	p.(Arg106Ser)	NA
23	31861	NA	f	29	MD	30	0.0	0.00	6	36	0.2	0.2	Y	EQUAL	P & M	N	Dense BSL	RCD	p.(Phe344Ser)	p.(Arg283Ter)	In trans
24	36021	Irish	f	23	CORD	55	0.2	0.3	5	60	0.5	0.6	Y	EQUAL	P & M	N	Diffuse BSL	RCD	Hom	p.(Arg283Ter)	NA
25	31391	Bengali	m	NA	CORD	49	0.5	1.00	8	57	1	HM	Y	EQUAL	P & M	Y (large)	None	R=C	Hom	p.(Arg106Ser)	NA
26	40330	Asian Indian	f	47	MD	47	0.5	0.3	16	63	1	1	Y	CENTRAL	P & M	N	Minimal BSL	R=C	p.(Arg283Ter)	p.(Arg364Ter)	NA
27	41209	NA	m	17	MD	18	0.5	0.60	3	21	1	1	N	CENTRAL	P & M	N	Minimal BSL	CRD	Hom	p.(Arg106Ser)	NA
28	39311	White British	f	25	MD	26	0.0	0.2	4	30	0.1	0.5	N	CENTRAL	N	NA	NA	MD	p.(Lys200Ter)	p.(Arg283Ter)	In trans
29	35483	Asian Indian	f	45	MD	44	0.2	0.2	6	50	0.3	0.5	Y	CENTRAL	N	Y	None	MD	p.(Met323ValfsTer20)	p.(Arg439Trp)	NA
30	41515	NA	m	12	MIXED	47	0.2	0.2	3	50	0.2	0.1	Y	CENTRAL	N	N	None	MD	Hom	p.(Asp526Asn)	NA
31	35925	Romanian	f	36	MIXED	38	0.5	0.8	7	45	0.5	NPL	Y	PERIPHERAL	P & M	N	Moderate BSL	NA	Hom	p.(Glu91Ter)	NA
32	39013	Asian Nepali	m	26	MIXED	28	0.2	0.2	3	31	0.6	0.6	N	PERIPHERAL	M	N	Minimal	NA	Hom	p.(Arg106Ser)	NA
33	28790	Asian Indian	m	30	MD	59	2.28	2.28	13	72	HM	HM	N	EQUAL	M	N	Minimal BSL	NA	p.(Arg106Ser)	p.(Arg106Cys)	NA
34e	44213	Black African	f	10	RCD	22	1.0	1.0	1	23	1.0	1.0	N	CENTRAL	NA	NA	NA	NA	p.Arg461Ter	c.613+2T>C	NA
35	39644	White British	f	20	MD	54	2.7	2.70	9	63	PL	PL	Y	EQUAL	N	N	None	NA	p.(Arg283Ter)	c.613+4_613+5delAG	NA
36	21364	NA	f	20	MD	58	2.28	2.28	16	74	NPL	NPL	N	EQUAL	P & M	N	Minimal	NA	c.1347-3C>G	p.(Arg283Ter)	NA
37g	37981	Asian Bangladeshi	f	16	MIXED	19	0.3	0.5	4	23	1.8	1.8	Y	CENTRAL	P & M	Y (large)	Moderate BSL	NA	Hom	p.(Arg283Ter)	NA
38g	46051	Asian Bangladeshi	f	15	ASYMPT.	15	0.3	0.50	4	19	0.30	0.50	N	CENTRAL	M	Y	None	NA	Hom	p.(Arg283Ter)	NA
39	5007	Asian Indian	m	14	CORD	42	2.28	2.28	13	55	PL	PL	N	EQUAL	N	Y (large)	None	NA	Hom	p.(Arg283Ter)	NA
40b	46582	Asian Pakistani	f	9	RCD	12	0.1	0.1	2	14	0.1	0.1	N	PERIPHERAL	P & M	N	None	NA	Hom	p.(Arg283Ter)	NA
41b	46583	Asian Pakistani	m	8	ASYMPT.	8	0.3	0.28	3	11	0.3	0.3	N	PERIPHERAL	N	Y	None	NA	Hom	p.(Arg283Ter)	NA
42d	NA	Irish	m	?	NA	32	1.98	0.80	16	48	PL	PL	NA	NA	NA	NA	NA	NA	Hom	p.(Arg283Ter)	NA
43	29879	White British	f	20	RCD	23	0.3	0.3	13	36	0.6	0.8	Y	EQUAL	N	N	None	NA	Hom	p.(Arg283Ter)	NA
44	NA	Irish	f	6	MD	21	0.5	0.5	0	NA	NA	NA	N	NA	M	N	Diffuse BSL	NA	Hom	p.(Arg283Ter)	NA
45	NA	Irish	m	20	MIXED	37	0.5	0.5	0	NA	NA	NA	NA	NA	NA	NA	Diffuse BSL	NA	Hom	p.(Arg283Ter)	NA
46	NA	Irish	m	13	RCD	21	0	0	37	58	NPL	NPL	NA	NA	N	NA	NA	NA	Hom	p.(Arg283Ter)	NA
47	3569	Asian Pakistani	m	15	RCD	67	2.7	2.70	9	76	PL	PL	N	PERIPHERAL	P & M	N	None	NA	Hom	p.(Arg283Ter)	NA

Abbreviations: NA: not applicable; y: years; m: male; f: female; Y: yes; N: no; RCD: rod-cone dystrophy; CORD: cone-rod dystrophy; MD: macular dystrophy; RE: right eye; LE: left eye; AF: autofluorescence; FU: follow up; HM: hand movements; PL: perception of light; P: peripheral; M: macular; CRA: chorio-retinal atrophy; BSL: bone-spicule-like; ERG: electroretinography; Hom: homozygous

Categories of presenting symptoms: RCD: night blindness, nyctalopia, peripheral vision loss; CORD: central vision loss, color vision issues; MD: central vision loss, photophobia, dysmorphosia.
