# Supplementary material for: CERKL-Associated Retinal Dystrophy: Genetics, Phenotype, and Natural History
Source: Ophthalmol Retina. 2023 Oct;7(10):918–31. doi: 10.1016/j.oret.2023.06.007 (PMC11108804; doi:10.1016/j.oret.2023.06.007)
Supplement: Supplementary Table 3 [file mmc2.pdf]

Variant No	Patient number	Nucleotide change (NM_001030311.2)	Amino acid change (NM_001030311.2)	Position	Coding impact (NM_001030311.2)	Location (NM_001030311.2)	Nucleotide change (NM_201548.5)	Amino acid change (NM_201548.5)	Coding impact (NM_201548.5)	Location (NM_201548.5)	dbSNP ID	ClinVar ID	Segregation
1	19	c.238+2T>C	NA	182521494	splice site alteration (canonical splice site)	intron 1 of 13 position 2 of 52689 (splicing-ACMG, splicing, intronic)	c.238+2T>C	NA	splice site alteration (canonical splice site)	intron 1 of 12 position 2 of 52689 (splicing-ACMG, splicing, intronic)	rs1436566598	Absent	
2	31	c.271G>T	p.Glu91Ter	182468774	nonsense	exon 2 of 14 position 33 of 243 (coding, NMD)	c.271G>T	p.Glu91Ter	nonsense	exon 2 of 13 position 33 of 243 (coding, NMD)	rs748876465	Absent	no segregation
3	11, 20, 22, 27, 32, 33	c.316C>A	p.Arg106Ser	182468729	missense	exon 2 of 14 position 78 of 243 (coding)	c.316C>A	p.Arg106Ser	missense	exon 2 of 13 position 78 of 243 (coding)	rs569826109	438054	
4	33	c.316C>T	p.Arg106Cys	182468729	missense	exon 2 of 14 position 78 of 243 (coding)	c.316C>T	p.Arg106Cys	missense	exon 2 of 13 position 78 of 243 (coding)	rs569826109	1308275	
5	14	c.557A>G	p.Tyr186Cys	182438536	missense	exon 3 of 14 position 76 of 132 (coding)	c.557A>G	p.Tyr186Cys	missense	exon 3 of 13 position 76 of 132 (coding)	NA	Absent	no segregation
6	28	c.598A>T	p.Lys200Ter	182438495	nonsense	exon 3 of 14 position 117 of 132 (coding, NMD)	c.598A>T	p.Lys200Ter	nonsense	exon 3 of 13 position 117 of 132 (coding, NMD)	rs398122963	91393	
7	17, 34	c.613+2T>C	NA	182438478	splice site alteration (canonical splice site)	intron 3 of 13 position 2 of 7631 (splicing-ACMG, splicing, intronic)	c.613+2T>C	NA	splice site alteration (canonical splice site)	intron 3 of 12 position 2 of 7631 (splicing-ACMG, splicing, intronic)	NA	Absent	no segregation
8	35	c.613+4_613+5del	NA	182438475	splice site alteration (intronic deletion)	intron 3 of 13 position 4-5 of 7631 (splicing, intronic)	c.613+4_613+5del	NA	splice site alteration (intronic deletion)	intron 3 of 12 position 4-5 of 7631 (splicing, intronic)	rs766131721	438055	
9	4	c.619G>T	p.Glu207Ter	182430843	nonsense	exon 4 of 14 position 6 of 64 (splicing, coding, NMD)	c.619G>T	p.Glu207Ter	nonsense	exon 4 of 13 position 6 of 64 (splicing, coding, NMD)	rs148109138	Absent	no segregation
10	1, 7, 8, 11, 13, 15, 16, 23, 24, 28, 29, 35 - 47	c.847C>T	p.Arg283Ter	182423344	nonsense	exon 6 of 14 position 92 of 143 (coding, NMD)	c.769C>T	p.Arg283Ter	nonsense	exon 5 of 13 position 92 of 143 (coding, NMD)	rs121909398	2364	segregation confirmed/no segregation done in either case
11	6, 21	c.941_942del	p.His314ArgfsTer55	182414392	frameshift	exon 7 of 14 position 43-44 of 75 (coding, NMD)	c.863_864del	p.His288ArgfsTer55	frameshift	exon 6 of 13 position 43-44 of 75 (coding, NMD)	NA	Absent	no segregation done in either case
12	23	c.1031T>C	p.Phe344Ser	182413527	missense	exon 8 of 14 position 58 of 178 (coding)	c.953T>C	p.Phe318Ser	missense	exon 7 of 13 position 58 of 178 (coding)	rs745341953	1056369	segregation confirmed
13	2, 9	c.1045_1046del	p.Met349ValfsTer20	182413512	frameshift	exon 8 of 14 position 72-73 of 178 (coding, NMD)	c.967_968del	p.Met323ValfsTer20	frameshift	exon 7 of 13 position 72-73 of 178 (coding, NMD)	rs750151209	236491	
14	3, 26	c.1090C>T	p.Arg364Ter	182413468	nonsense	exon 8 of 14 position 117 of 178 (coding, NMD)	c.1012C>T	p.Arg338Ter	nonsense	exon 7 of 13 position 117 of 178 (coding, NMD)	rs748394238	280382	no segregation done in either case
15	10, 18	c.1151+3_1151+6del	NA	182413401	splice site alteration (intronic deletion)	intron 8 of 13 position 3-6 of 75 (splicing, intronic)	c.1073+3_1073+6del	NA	splice site alteration (intronic deletion)	intron 7 of 12 position 3-6 of 75 (splicing, intronic)	rs1553513437	438052	
16	6, 21	c.1238-1G>A	NA	182412454	NA	intron 10 of 13 position 95 of 95 (splicing-ACMG, splicing, intronic)	c.1160-1G>A	NA	NA	intron 9 of 12 position 95 of 95 (splicing-ACMG, splicing, intronic)	NA	Absent	segregation confirmed
17	36	c.1347-3C>G	NA	182409526	splice site alteration (intronic change)	intron 11 of 13 position 2819 of 2821 (splicing, intronic)	c.1269-3C>G	NA	splice site alteration (intronic change)	intron 10 of 12 position 2819 of 2821 (splicing, intronic)	rs1553512879	438053	no segregation
18	5, 17, 34	c.1381C>T	p.Arg461Ter	182409489	nonsense	exon 11 of 13 position 35 of 97 (coding, NMD)	c.1303C>T	p.Arg435Ter	nonsense	exon 12 of 14 position 35 of 97 (coding, NMD)	rs1187839124	555316	no segregation
19	29	c.1393C>T	p.Arg465Trp	182409477	missense	exon 12 of 14 position 47 of 97 (coding)	c.1315C>T	p.Arg439Trp	missense	exon 11 of 13 position 47 of 97 (coding)	rs139300604	855128	
20	30	c.1576G>A	p.Asp526Asn	182403859	missense	exon 13 of 14 position 133 of 173 (coding)	c.1498G>A	p.Asp500Asn	missense	exon 12 of 13 position 133 of 173 (coding)	rs771072583	809116	no segregation
21	12	c.1644T>G	p.Tyr548Ter	182402944	nonsense	exon 14 of 14 position 28 of 1571 (coding)	c.1566T>G	p.Tyr522Ter	nonsense	exon 13 of 13 position 28 of 1573 (coding)	rs1278226911	866584	segregation confirmed in trans
22	19	Exon 1 deletion			large deletion (exon deletion)	deletion of exon 12	exon 1 deletion		large deletion (exon deletion)	deletion of exon 12	NA	Absent	
23	5	Exon 2 deletion			large deletion (exon deletion)	deletion of exon 12	exon 2 deletion		large deletion (exon deletion)	deletion of exon 12	NA	Absent	

Variant No	ACMG Classification						GnomAD allele frequency													
	Verdict	Idntified classification rules					Allele frequency (exome)						Allele frequency (genome)					Coverage in gnomAD Exomes samples		
		Factor 1	Factor 2	Factor 3	Factor 4	Factor 5	East Asian	South Asian	African	European (Non-Finnish)	Latino	Total	East Asian	South Asian	African	European (Non-Finnish)	Total	Mean coverage	Median coverage	% of samples over 20x coverage
1	Pathogenic	PVS1	PM2	PM3			0	0	0	0	0	0	NA	NA	NA	NA	NA	20	19	47.39%
2	Likely pathogenic	PVS1	PM2				0	0	0	8.88E-06	0	4.01101E-06	NA	NA	NA	NA	NA	43.3	39	90.53%
3	Likely pathogenic	PM2	PP1	PP3	PP5		0	0.000426	0	0	0	5.20391E-05	NA	NA	NA	NA	NA	53.6	53	93.97%
4	VUS	PM2	PM3	PP5			5.48E-05	0.000164	0	3.55E-05	2.9E-05	5.20391E-05	0	0	0.000115	6.48E-05	9.56E-05	53.6	53	93.97%
5	VUS	PM2	PP3				NA	NA	NA	NA	NA	NA	NA	NA	NA	NA	NA	56	53	95.33%
6	Pathogenic	PVS1	PM2	PM3	PP5		0	0	0	8.83E-06	0	3.99323E-06	NA	NA	NA	NA	NA	47.2	41	95.01%
7	Likely pathogenic	PVS1	PM2				NA	NA	NA	NA	NA	NA	NA	NA	NA	NA	NA	42.1	36	93.91%
8	VUS	PM2	PM3	PP5			0	0	0	1.77E-05	0	7.99635E-06	NA	NA	NA	NA	NA	40.8	35	93.13%
9	Likely pathogenic	PVS1	PM2				0	0	0	0	0	0	NA	NA	NA	NA	NA	58.2	54	98.75%
10	Pathogenic	PVS1	PM2	PM3	PP5		0	0.000523	0.000129	0.00055	0.000174	0.000345615	0	0	0	0.000454	0.000223	87.8	100	99.67%
11	Likely pathogenic	PVS1	PM2				NA	NA	NA	NA	NA	NA	NA	NA	NA	NA	NA	72.1	73	99.53%
12	VUS	PM2	PM3				0	0	0	7.06E-05	0	0.000035866	0	0	0	6.48E-05	3.18E-05	84.5	91	100%
13	Pathogenic	PVS1	PM2	PP1	PM3	PP5	0	9.8E-05	0	0	0	1.19549E-05	NA	NA	NA	NA	NA	87.1	97	99.77%
14	Pathogenic	PVS1	PM2	PP5			0	3.27E-05	0	8.82E-06	2.89E-05	1.19537E-05	0	0	0.00023	0	6.37E-05	87.8	97	100%
15	Likely pathogenic	PVS1	PM2	PP1	PP5		NA	NA	NA	NA	NA	NA	NA	NA	NA	NA	NA	80.8	89	100%
16	Likely pathogenic	PVS1	PM2				NA	NA	NA	NA	NA	NA	NA	NA	NA	NA	NA	98.4	100	100%
17	VUS	PM2	PM3	PP5			NA	NA	NA	NA	NA	NA	NA	NA	NA	NA	NA	29.7	27	70.17%
18	Pathogenic	PVS1	PM2	PM3	PP5		0	3.36E-05	0	8.86E-06	0	8.03697E-06	0	0	0.000115	0	3.19E-05	38.2	34	87.96%
19	VUS	PM2	PM3	PP5			5.48E-05	6.66E-05	0	3.54E-05	0.000117	0.000044102	NA	NA	NA	NA	NA	38.4	34	87.15%
20	VUS	PM2	PP3				0	0.000163	0	1.76E-05	2.89E-05	3.18504E-05	NA	NA	NA	NA	NA	78	79	99.85%
21	VUS	PVS1 moderate	PM2	PP5			0	0	0	2.66E-05	0	1.20187E-05	NA	NA	NA	NA	NA	48.9	48	95.95%
22	Pathogenic	PVS1	PM2	PM3			NA	NA	NA	NA	NA	NA	NA	NA	NA	NA	NA	NA	NA	NA
23	Pathogenic	PVS1	PM2	PM3			NA	NA	NA	NA	NA	NA	NA	NA	NA	NA	NA	NA	NA	NA

Variant No	General predisction																
	MutationTaster			FATHMM			FATHMM MKL			FATHMM XF			CADD	REVEL		
	Prediction	Accuruacy	Converted rankscore	Prediction	Score	Converted rankscore	Prediction	Score	Converted rankscore	Prediction	Score	Converted rankscore	Score	Prediction	Score	Rankscore
1	Disease causing	1	0.81001	NA	NA	NA	Damaging	0.93589	0.58631	NA	NA	NA	33	NA	NA	NA
2	Disease causing automatic	1	0.81001	NA	NA	NA	Damaging	0.97036	0.72319	Neutral	0.127858	0.24539	43	NA	NA	NA
3	Disease causing	0.999996	0.58761	Tolerated	2.14	0.40469	Damaging	0.9946	0.96305	Damaging	0.953484	0.96982	26.8		0.374	
4	Disease causing	1	0.81001	Tolerated	2.05	0.42122	Damaging	0.99396	0.95489	Damaging	0.95417	0.97107	32		0.286	
5	Disease causing	1	0.81001	Tolerated	2.5	0.14408	Damaging	0.96059	0.67346	Damaging	0.92579	0.90588	25.6		0.499	
6	Disease causing automatic	1	0.81001	NA	NA	NA	Damaging	0.76043	0.37266	Neutral	0.30278	0.41093	39	NA	NA	NA
7	Disease causing	1	0.81001	NA	NA	NA	Damaging	0.934	0.58116	NA	NA	NA	32	NA	NA	NA
8	NA	NA	NA	NA	NA	NA	NA	NA	NA	NA	NA	NA	15.14	NA	NA	NA
9	Disease causing automatic	1	0.81001	NA	NA	NA	Damaging	0.96623	0.70072	Neutral	0.134368	0.25436	38	NA	NA	NA
10	Disease causing automatic	1	0.81001	NA	NA	NA	Neutral	0.02108	0.06242	Neutral	0.105401	0.21061	32	NA	NA	NA
11	NA	NA	NA	NA	NA	NA	NA	NA	NA	NA	NA	NA	35	NA	NA	NA
12	Disease causing	0.999969	0.52935	Tolerated	2.14	0.1945	Damaging	0.97493	0.75105	Damaging	0.946778	0.9566	29.6		0.459	
13	NA	NA	NA	NA	NA	NA	NA	NA	NA	NA	NA	NA	35	NA	NA	NA
14	Disease causing automatic	1	0.81001	NA	NA	NA	Damaging	0.9602	0.67171	Neutral	0.277698	0.39215	41	NA	NA	NA
15	NA	NA	NA	NA	NA	NA	NA	NA	NA	NA	NA	NA	14.75	NA	NA	NA
16	Disease causing	1	0.81001	NA	NA	NA	Damaging	0.98567	0.84178	NA	NA	NA	34	NA	NA	NA
17	NA	NA	NA	NA	NA	NA	NA	NA	NA	NA	NA	NA	18.02	NA	NA	NA
18	Disease causing automatic	1	0.81001	NA	NA	NA	Damaging	0.91736	0.54154	Neutral	0.280265	0.39413	38	NA	NA	NA
19	Disease causing	0.999987	0.58761	Tolerated	2.43	0.15261	Damaging	0.95525	0.65068	Damaging	0.922677	0.89802	33		0.39	
20	Disease causing	0.999995	0.58761	Tolerated	-0.47	0.70133	Damaging	0.96284	0.68389	Damaging	0.86203	0.78023	31		0.71	
21	Disease causing	1	0.81001	NA	NA	NA	Damaging	0.94316	0.60778	Neutral	0.090611	0.18359	47	NA	NA	NA
22	NA	NA	NA	NA	NA	NA	NA	NA	NA	NA	NA	NA	NA	NA	NA	NA
23	NA	NA	NA	NA	NA	NA	NA	NA	NA	NA	NA	NA	NA	NA	NA	NA

Variant No	Functional prediction								Human Splice Finder 3.0	Conservation				Conservation				Report name	Year	Report name	Year	Novel variants
	SIFT			PROVEAN			Polyphen2			PhyloP30way		PhastCons30way		PhyloP100way		PhastCons100way						
	Prediction	Score	Converted rankscore	Prediction	Score	Converted rankscore	Prediction	Score		Mammalian	Mammalian rankscore	Mammalian	Mammalian rankscore	vertebrate	vertebrate rankscore	vertebrate	vertebrate rankscore					
1	NA	NA	NA	NA	NA	NA	NA	NA		1.312	0.94714	0.646	0.29799	4.72	0.61636	1	0.71638	Ellingford (2018) J Med Genet 55, 114	2018			
2	NA	NA	NA	NA	NA	NA	NA	NA		1.026	0.45946	0.997	0.62031	4.576	0.60626	1	0.71638	NA				Novel
3	Damaging	0.099,0.	0.41865	Damaging	-3.48	0.70067	Probably damaging	0.997		1.176	0.78918	0.983	0.48746	7.847	0.85186	1	0.71638	Ali (2008) Mol Vis 14, 1960	2008	Downes (2020) Genes (Basel) 11: [Additional phenotype]	2020	
4	Damaging	0.004	0.91219	Damaging	-5.2	0.83695	Probably damaging	0.997		1.176	0.78918	0.983	0.48746	7.847	0.85186	1	0.71638	Downes (2020) Genes (Basel) 11: [Additional phenotype]		Avela (2018) Acta Ophthalmol 96: 183 [Additional phenotype]	2018	
5	Damaging	0	0.91219	Damaging	-6.34	0.91522	Probably damaging	0.999		1.138	0.64695	0.123	0.20176	6.175	0.71875	1	0.71638	NA				Novel
6	NA	NA	NA	NA	NA	NA	NA	NA		0.2	0.23007	0.997	0.62031	1.459	0.34844	1	0.71638	Nishiguchi (2013) Proc Natl Acad Sci U S A 110, 16139	2013	Downes (2020) Genes (Basel) 11: [Additional phenotype]	2020	
7	NA	NA	NA	NA	NA	NA	NA	NA		1.312	0.94714	0.976	0.46477	7.169	0.77141	1	0.71638	NA				Novel
8	NA	NA	NA	NA	NA	NA	NA	NA		NA	NA	NA	NA	NA	NA	NA	NA	Carss (2017) Am J Hum Genet 100, 75	2017	Downes (2020) Genes (Basel) 11: [Additional phenotype]	2020	
9	NA	NA	NA	NA	NA	NA	NA	NA		1.026	0.45946	0.84	0.34853	2.701	0.46761	0.989	0.36753	NA				Novel
10	NA	NA	NA	NA	NA	NA	NA	NA		-1.079	0.02227	0.705	0.31045	-0.078	0.11338	0	0.06391	Downes (2020) Genes (Basel) 11: [Additional phenotype]	2020	Avela (2018) Acta Ophthalmol 96: 183 [Additional phenotype]	2018	
11	NA	NA	NA	NA	NA	NA	NA	NA		NA	NA	NA	NA	NA	NA	NA	NA	NA				Novel
12	Damaging	0.001	0.78421	Damaging	-5.79	0.88431	Probably damaging	0.953		1.312	0.94714	1	0.86279	7.157	0.7705	1	0.71638	NA				Novel
13	NA	NA	NA	NA	NA	NA	NA	NA		NA	NA	NA	NA	NA	NA	NA	NA	Downes (2020) Genes (Basel) 11: [Additional phenotype]	2020	Wang (2017) BMC Ophthalmol 17: 157 [Additional phenotype]	2017	
14	NA	NA	NA	NA	NA	NA	NA	NA		1.176	0.78918	0.996	0.59523	6.12	0.71312	1	0.71638	Wang (2017) BMC Ophthalmol 17: 157 [Additional phenotype]	2017	Downes (2020) Genes (Basel) 11: [Additional phenotype]	2020	
15	NA	NA	NA	NA	NA	NA	NA	NA		NA	NA	NA	NA	NA	NA	NA	NA	Habibi (2016) Sci Rep 6, 37455	2016	Downes (2020) Genes (Basel) 11: [Additional phenotype]	2020	
16	NA	NA	NA	NA	NA	NA	NA	NA		1.026	0.45946	0.997	0.62031	5.122	0.64536	1	0.71638	NA				Novel
17	NA	NA	NA	NA	NA	NA	NA	NA		NA	NA	NA	NA	NA	NA	NA	NA	Downes (2020) Genes (Basel) 11: [Additional phenotype]	2020	Turro (2020) Nature 583: 96 [Additional report]	2020	
18	NA	NA	NA	NA	NA	NA	NA	NA		0.23	0.25697	0.984	0.49151	3.072	0.49747	1	0.71638	Downes (2020) Genes (Basel) 11: [Additional phenotype]	2020	Ellingford (2018) J Med Genet 55: 114 [Additional phenotype]	2018	
19	Damaging	0.001	0.91219	Damaging	-7.05	0.93856	Probably damaging	0.999		1.176	0.78918	0.975	0.4621	5.198	0.64983	1	0.71638	Downes (2020) Genes (Basel) 11, 1497	2020			
20	Damaging	0.007	0.65363	Damaging	-4.32	0.76655	Probably damaging	1		1.026	0.45946	1	0.86279	6.221	0.72188	1	0.71638	NA				Novel
21	NA	NA	NA	NA	NA	NA	NA	NA		1.312	0.94714	1	0.86279	4.663	0.61226	1	0.71638	NA				Novel
22	NA	NA	NA	NA	NA	NA	NA	NA		NA	NA	NA	NA	NA	NA	NA	NA	Ellingford (2018) J Med Genet 55, 114	2018			
23	NA	NA	NA	NA	NA	NA	NA	NA		NA	NA	NA	NA	NA	NA	NA	NA	Birtel (2018) Sci Rep 8	2018			

Genome reference; Hg38 Transcript; NM_001030311.2, ENST00000339098.9 or NM_201548.5, ENST00000410087.8

Supplementary Table 3: Analysis of variants present in our cohort.

Footnote 1. Sequence variant nomenclature was obtained according to the guidelines of the Human Genome Variation Society (HGVS) by using Mutalyzer (https://mutalyzer.nl/). All detected variants were analysed using two general prediction programs and three functional prediction programs; Mutation Taster (http://www.mutationtaster.org/), FATHMM (http://fathmm.biocompute.org.uk/9), SIFT (https://www.sift.co.uk/), PROVEAN (http://provean.jcvi.org/index.php), and Polyphen 2 (http://genetics.bwh.harvard.edu/pph2/). Minor allele frequency for the identified variants in the general population was assessed in the Genome Aggregation Database (gnomAD) datasets(https://gnomad.broadinstitute.org/). The population data and general coverage were also provided with the gnomAD database. Evolutionary conservation scores were calculated referring to the UCSC database (https://genome.ucsc.edu/index.html). Classification of predictions by the American College of Medical Genetics and Genomics (ACMG) was also applied for all detect
databases were accessed on 1 November 2022. Pathogenicity classification was assessed according to the following factors: PVS1 (null variant (nonsense, frameshift, canonical ±1 or 2 splice sites, initiation codon, single or multiexon deletion) in a gene where loss of function is a known mechanism of disease); PS2 (pathogenicity supportive 2; de novo (both maternity and paternity confirmed) in a patient with the disease and no family history); PS3 (pathogenicity supportive 3; well-established in vitro or in vivo functional studies supportive of a damaging effect on the gene or gene product); PM1 (pathogenicity moderate 1; located in a mutational hot spot and/or critical and well-established functional domain without benign variation); PM2 (pathogenicity moderate 2; absent or very rare from controls in Exome Sequencing Project, 1000 Genomes Project, or Exome Aggregation Consortium); PM5 (pathogenicity moderate 5; novel missense change at an amino acid residue where a different missense change determined to be p
PP1 (pathogenicity supporting 1; co-segregation with disease in multiple affected family members in a gene definitively known to cause the disease); PP3 (pathogenicity supporting 3; multiple lines of computational evidence support a deleterious effect on the gene or gene product); PP5 (pathogenicity supporting 5; reputable source recently reports variant as pathogenic, but the evidence is not available from the laboratory to perform an independent evaluation). All databases were accessed on 1 November 2022.
