# Supplementary material for: CERKL-Associated Retinal Dystrophy: Genetics, Phenotype, and Natural History
Source: Ophthalmol Retina. 2023 Oct;7(10):918–31. doi: 10.1016/j.oret.2023.06.007 (PMC11108804; doi:10.1016/j.oret.2023.06.007)
Supplement: Supplementary Table 4 [file mmc3.pdf]

Patients (probands)	Phenotype	Symptoms	Most common Retinal features	VA range	ERG	OCT	Country (ethnicity)	Author (year)
6 (6)	4 RP & 2 MD	Central vision loss and nyctalopia (7-45)	Central atrophy and chorioretinal atrophic patches	6/12 to NPL	Reduced cone and rod amplitudes	Outer retinal loss	UK (white and Asian)	Downes et al (2020)[1]
14 (14)	RP	Central vision loss and nyctalopia (5–55)	Mild to moderate disc pallor, paucipigmentary character, early macular involvement	0.1 to 3 LogMAR	Reduced photopic and scotopic responses	Outer retinal atrophy	India	Sen et al (2020)[2]
3 (1)	RP	Central vision loss, nyctalopia and field constriction	Severe degeneration of the peripheral and central retina, including the macula	-	Non detectable as early as 8 yo	-	Pakistan	Nadeem et al (2020)[3]
9 (2)	CORD	Decreased VA (12 – 18)	Attenuated blood vessels, bony spicules, and macular degeneration.	CF to LP	Severely reduced ffERG	Central and para-central atrophy	Jordan	Azab et al (2019)[4]
4 (4)	CORD	Progressive loss of visual acuity in all, with nyctalopia in 2 (age NA)	Extensive maculopathy and retina-wide degeneration, with hyperautofluorescent foci adjacent to the macular atrophy	20/40 to hand motion	Extinguished rod-specific b-wave, residual flicker in 3	extensive atrophy of the outer retinal lamina	USA (Irish, Indian and Greek)	Sengillo et al (2019)[5]
18 (18)	CORD & MD	Night blindness (7 – 38)	Macular RPE granularity	0.2 to LP	Diminished rod and subnormal cone responses	Macular thinning of RPE and outer retina	Finland	Avela et al (2018)[6]
2 (1)	RP	Nyctalopia and central vision loss (late teens to early 20s)	Bilateral perifoveal RPE atrophy, and diffuse RPE pigmentary mottling extended throughout the fundus	20/40 to 20/63	Not measurable scotopic; reduced cone responses	Outer retinal atrophy	USA (India)	Biswas et al (2017)[7]
4 (4)	RP	Nyctalopia and photophobia (4 – 25)	Few pigment deposits mild optic disc and peripheral atrophy	5/10 to 1/10	-	-	Tunisia	Habibi et al (2016)[8]
1 (1)	RP	Decreased acuity and photophobia (16 yo)	Pale optic disc, slightly retina vessels attenuation and extensive RPE macular atrophy well delimited	0.2/0.2 LogMAR	Pathologic flash both eyes	-	Spain	Sánchez-Alcudia et al (2014)[9]
3 (2)	CORD	Symptom NA (28-30)	Macular atrophy	20/50 to LP	Severely reduced or abolished rod and cone responses	Retinal and RPE thinning at the posterior pole	The Netherlands (Canada)	Littink et al (2010)[10]
3 (1)	RP	Night blindness (18 -20)	Attenuation of retinal arteries, bony spicule pigmentation, normal colour of optic discs, bull's-eye macula	HM	-	-	China	Tang et al (2009)[11]

6 (3)	CORD	Reading difficulties - night vision disturbances	macular RPE depigmentation or atrophy, and a diffuse granular appearance of the more peripheral retina. In the more severely affected patients, there was extensive central chorioretinal atrophy and peripheral pigmentary retinopathy.	20/25 to LP	Greater cone than rod dysfunction.	Thinned fovea, reduced foveal ONL, patches of hyperreflectivity, inner laminopathy.	USA (Europe)	Aleman et al (2009)[12]
5 (1)	-	Nyctalopia and field loss (early teens)	Disc pallor, arterial attenuation, bone spicule pigmentation anterior and posterior to the equator, cellophane maculopathy	PL and NPL	-	-	UK (Pakistan)	Ali et al (2008)[13]
9 (7)	RP	Nyctalopia and progressive loss of VA (13-34)	Peripheral bone spicules, well-demarcated areas of chorioretinal atrophy; macular atrophy with annular hyperpigmentation	20/50 to LP	Not detectable	-	Spain	Avila-Fernandez et al (2008)
25 (7)	-	Mild night and central vision impairment (teen years)	Widespread macular and peripheral atrophy, with pigmentary changes	6/7.5 to HM	Similar degree of rod and cone dysfunction	-	Israel (Yemen)	Auslender et al (2007)[14]

Supplementary Table 4. Literature review of *CERKL*-associated retinopathy cases. RP: retinitis pigmentosa; CORD: cone-rod dystrophy; MD: macular dystrophy; VA: visual acuity; NA: not available; HM: hand movements; PL: perception of light; NPL: no perception of light; RPE: retinal pigment epithelium; ERG: electroretinography; ffERG: full-field electroretinography; OCT: optical coherence tomography; ONL: outer nuclear layer.
