# Supplementary material for: CERKL-Associated Retinal Dystrophy: Genetics, Phenotype, and Natural History
Source: Ophthalmol Retina. 2023 Oct;7(10):918–31. doi: 10.1016/j.oret.2023.06.007 (PMC11108804; doi:10.1016/j.oret.2023.06.007)
Supplement: fig 7 [file mmc4.pdf]

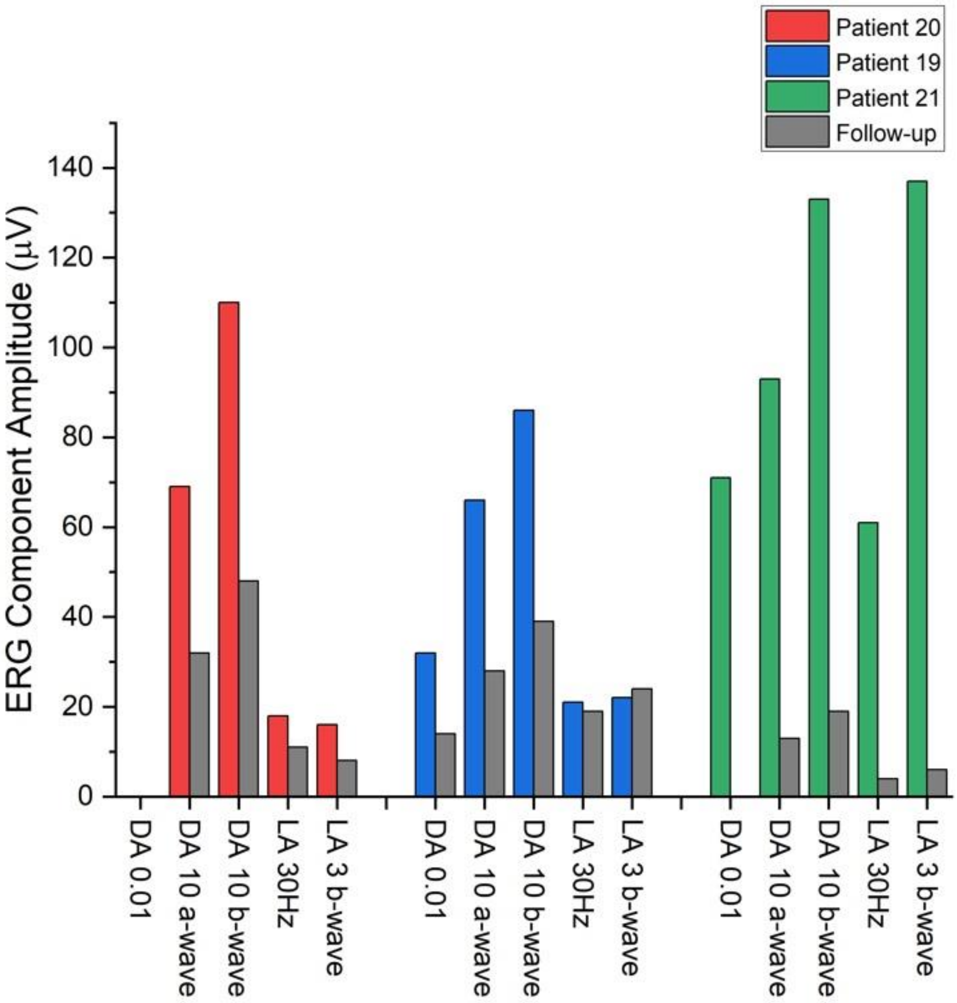

Supplemental figure 7. Comparison of the main ERG and PERG P50 amplitude parameters at baseline with those obtained at follow-up for patients 19, 20 and 21. See text for details.
