# Supplementary material for: CERKL-Associated Retinal Dystrophy: Genetics, Phenotype, and Natural History
Source: Ophthalmol Retina. 2023 Oct;7(10):918–31. doi: 10.1016/j.oret.2023.06.007 (PMC11108804; doi:10.1016/j.oret.2023.06.007)
Supplement: fig 8 [file mmc5.pdf]

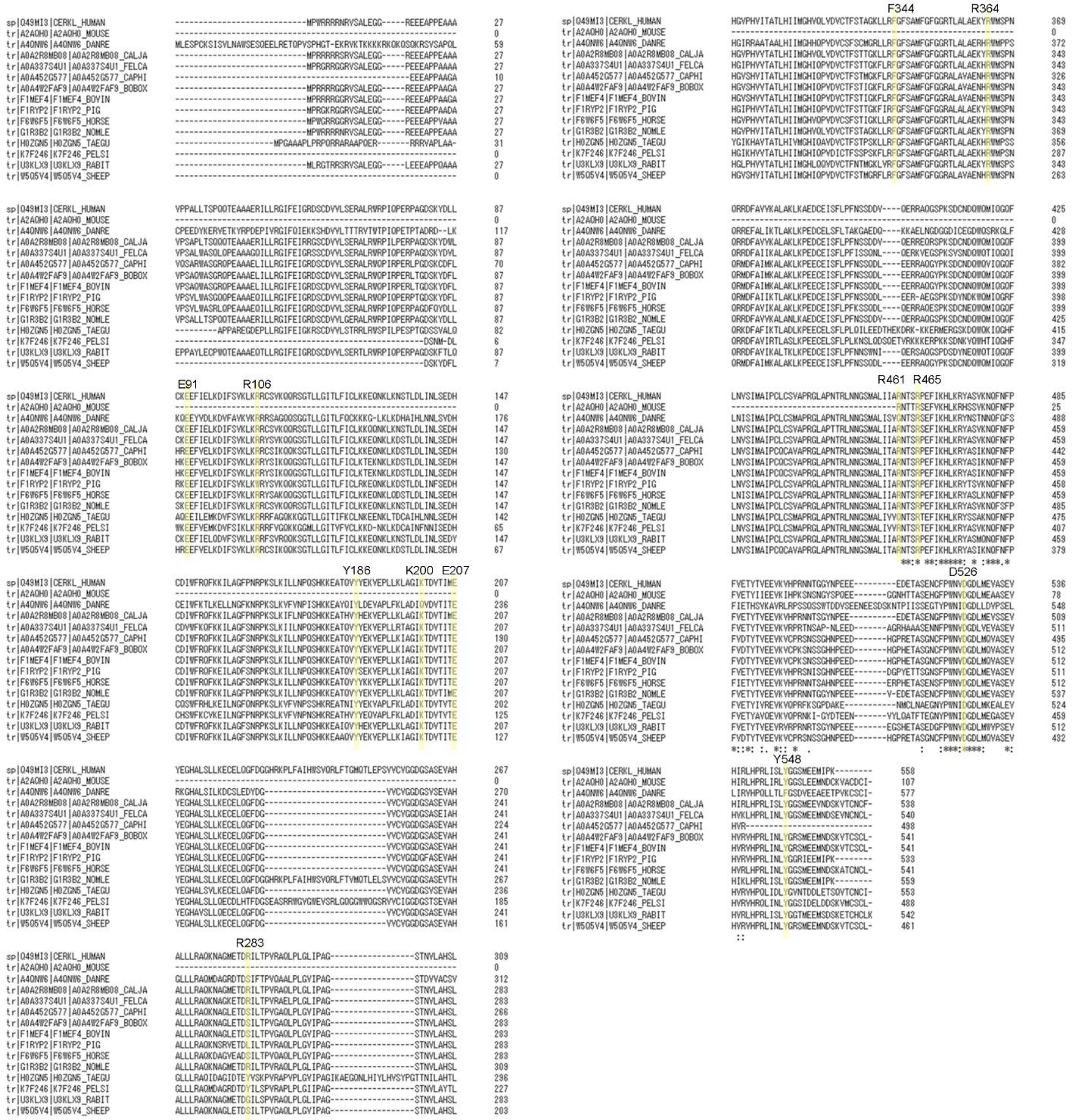

Supplementary figure 9. Multiple alignment of 15 species of CERKL. The alignment was performed with the Clustal Omega program (https://www.ebi.ac.uk/Tools/msa/clustalo/;accessed on 1 November 2022). The amino acid-sequence alignment is numbered in accordance with the Homo sapiens CERKL sequence (Uniprot: Q49MI3, ENSG00000188452, ENST00000339098.9). An asterisk indicates perfect conservation across the species. The positions of variant residues are highlighted with a yellow background: c.271G>T (p.Glu91Ter), c.316C>A (p.Arg106Ser), c.316C>T (p.Arg106Cys), c.557A>G (p.Tyr186Cys), c.598A>T (p.Lys200Ter), c.619G>T (p.Glu207Ter), c.847C>T (p.Arg283Ter), c.1031T>C (p.Phe344Ser), c.1090C>T (p.Arg364Ter), c.1381C>T (p.Arg461Ter), c.1393C>T (p.Arg465Trp), c.1576G>A (p.Asp526Asn), c.1644T>G (p.Tyr548Ter).
